# Microbial Source Tracking Approach to Investigate Fecal Waste at the Strawberry Creek Watershed and Clam Beach, California, USA

**DOI:** 10.3390/ijerph18136901

**Published:** 2021-06-27

**Authors:** Jeremy A. Corrigan, Steven R. Butkus, Michael E. Ferris, Jill C. Roberts

**Affiliations:** 1 Humboldt County Public Health Laboratory, County of Humboldt, 529 I Street, Eureka, CA 95501, USA; micromikepetaluma@gmail.com; 2 College of Public Health, University of South Florida, 13201 Bruce B. Downs Blvd, MDC 56, Tampa, FL 33612, USA; jcrobert@usf.edu; 3 State of California, North Coast Regional Water Quality Control Board, 5550 Skylane Blvd., Santa Rosa, CA 95403, USA; stevebutkus@yahoo.com

**Keywords:** bird, canine, fecal indicator bacteria, human, recreational water, ruminant

## Abstract

Clam Beach is located in Northern California, USA, and is listed as an impaired waterway by the federal government. The scope of this study was to investigate this beach and surrounding watershed to determine, if possible, the source of the impairment by conducting an 11-h beach study and 8-week watershed study. We used traditional fecal indicator bacteria (FIB) and microbial source tracking (MST) methods to help identify source(s) of the FIB. Our study was focused on four possible contributors: human, ruminant, canine, and bird. A total of 169 samples were collected, analyzed, and compared to the California Department of Health single sample maximum (SSM) objective. In the beach study, 29 (44%) samples exceeded at least one SSM objective, which would have resulted in a resample per state regulations for recreational primary contact use. MST methods showed that the most abundant marker detected was bird, in 65% of the samples, but varied by sample location, which is likely due to a natural population of nearshore birds regularly observed along Clam Beach. The watershed study highlighted the potential influence from ruminants throughout the region, while humans did not appear to be a significant contributor. Health risk to humans appears to be low.

## 1. Introduction

Recreational waterways are frequent leisure destinations for families and important economic resources for many coastal communities [1]. Keeping visitors safe from harmful water pathogens and preventing adverse health outcomes is an important public health focus. Heavy rains can cause a significant rise in water level of creeks and streams, potentially carrying high levels of bacteria and other pollutants to the ocean, adding potential sources of fecal indicator bacteria (FIB) and surface runoff [2]. These bacteria and pollutants may be present at recreational waterways and can cause recreational water illness (RWI). RWI is caused by swimmers swallowing water that is potentially contaminated by a variety of pathogens that can cause diarrhea, skin disease, or other potentially serious illness [3]. In the United States and other countries, *E. coli* (EC) and enterococci (ENT) bacteria, commonly called FIB, are often used to regulate beaches [4,5]. The United States Environmental Protection Agency (USEPA) recently published new guidelines for EC and ENT that aim to keep the risk of gastrointestinal (GI) illness in swimmers below approximately 30 illnesses per 1000 swimmers (2012 RWQC) [5]. In addition, there are epidemiology studies that link FIB to illnesses in swimmers at coastal beaches [6]. Current protocols for monitoring and alerting the public of adverse water conditions to protect human health are limited, i.e., sample variability, no source determination, and 24 h for results. However, new laboratory methods are being developed and their use continues to show promise in adding depth to the current testing methodologies [7,8].

There are many potential sources for fecal waste contamination in recreational waterways, from both human and non-human sources [9]. The risks associated with these sources can fluctuate with environmental conditions such as rainfall. Storm water runoff can introduce animal fecal contamination. However, the risk to human health is often an order of magnitude lower than if it were from a human source [10]. Gulls have also been identified as a potential source of fecal contamination, which can often be variable due to migration and the varied mobility of gull populations. The transient nature of gulls poses a difficult challenge as they have been shown to successfully transport fecal contamination from human waste sites and transport bacteria to beaches, thus increasing the risk to visitors [11]. Dogs are also frequent visitors to beaches and contribute to fecal contamination [12]. These studies show that fecal contamination can be complex and mixed with both human and non-human fecal sources, increasing potential risk of illness to visitors.

FIB such as EC and ENT are variable in monitoring recreational water and have limited capacity to discern human from non-human sources [4]. Despite their limitations, detection of FIB remains the gold standard in recreational water monitoring. Determining the source of measured spikes of FIB and correlating them with an epidemiological study allow for better understanding of recreational waterways and possible links to adverse health outcomes [13]. Microbial source tracking (MST) describes a suite of methods and investigative strategy to determine the source of fecal contamination in environmental water that depends on the association of certain fecal microorganisms with a particular host. MST field studies are being performed with more frequency to help identify the source of fecal contamination [12,14]. These methods can detect host-associated *Bacteroides* bacteria or other microorganisms from humans and other animals and link them to GI illness in recreational water [15,16]. Inter-laboratory variation has been identified within MST studies; therefore, standardization of protocols and consumable reagents is recommended for repeatability and reproducibility [17]. Few public health laboratories have fully adopted MST methods as part of routine beach management strategies, even though they show promise in helping control hazards to beachgoers.

The federal Clean Water Act (CWA) is the law that regulates pollution of United States waterways. Section 303(d) of the CWA requires states like California to identify surface waters where currently applied pollution control technologies cannot meet the water quality standards of the waterbody [18]. Clam Beach is listed on the Section 303(d) list of impaired waterways for high bacteria levels. Clam Beach commonly exceeds the single sample maximum (SSM) for beach closures and advisories conducted by the Department of Health Services [19]. Additionally, Clam Beach near Strawberry Creek was selected for this project because FIB levels have recently given this popular beach a grade of “F” on the Heal the Bay’s Annual Beach Report Card, a ranking of the 10 most polluted beaches in the State and earned the No. 2 spot on California’s Top Ten Beach Bummers List, up from the No. 4 spot the previous year [20]. The greater Strawberry Creek watershed, comprised of Patrick Creek, Strawberry Creek, and Rose Creek, directly influences the density and potential source(s) of FIB levels at Clam Beach. This region has a variety of land uses that include agricultural, industrial, timberlands, public, and residential, all of which potentially influence fecal waste at Clam Beach. As part of a pilot project, during the dry weather beach monitoring season, which is April through October annually, a total of 91 FIB and 103 MST samples were collected and analyzed at Clam Beach from August 2015 to April 2018, which showed that birds were the most likely source of fecal contamination (Table S1). The study presented here provides further insight into the potential source(s) and distribution of fecal waste at Clam Beach and Strawberry Creek watershed by conducting a beach study and watershed study. Additionally, it attempts to integrate these MST methods into the daily public health laboratory workflow and beach monitoring protocols.

## 2. Materials and Methods

### 2.1. Study Location and Watershed Sanitary Survey

**Figure 1:**
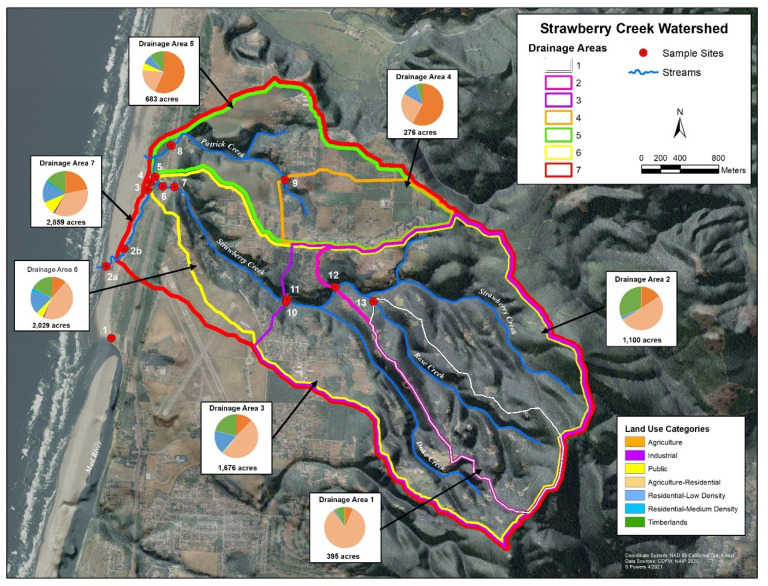
GIS map of the study area showing the 14 collection sites along Clam Beach, Strawberry Creek, Patrick Creek, and Rose Creek used in this study. A description of the seven drainage areas, land use composition, and relative size is shown. County of Humboldt GIS.

Clam Beach is located in northern California, USA, and is directly influenced by the Strawberry Creek watershed. Results of the sampling survey identified seven drainage areas with a variety of land uses, which are shown in Figure 1. Additionally, the primary land use by area is shown in Figure S1. Each sampling site is influenced by a variety of potential sources, which are summarized in Table S2. Sample site 1 is a beach monitoring sampling site at Clam Beach near Mad River and was chosen as a comparison site because it is not considered impaired. Sample site 2a is also a beach monitoring site at Clam Beach near Strawberry Creek. This site is considered impaired and is the primary focus of this study. Sample site 2b represents Strawberry Creek right before it meets Clam Beach. Sample sites 3–7 represent the headwaters of Strawberry Creek with site 5 having the most human and dog activity. Sites 8 and 9 represent the Patrick Creek watershed with residential and agriculture influences. Sites 10–11 represent upstream Strawberry Creek with the most potential for septic system influence. Site 12 is furthest upstream Strawberry Creek, with site 13 on Rose Creek as it meets Strawberry Creek.

### 2.2. Beach and Watershed Study Collection

**Figure 2:**
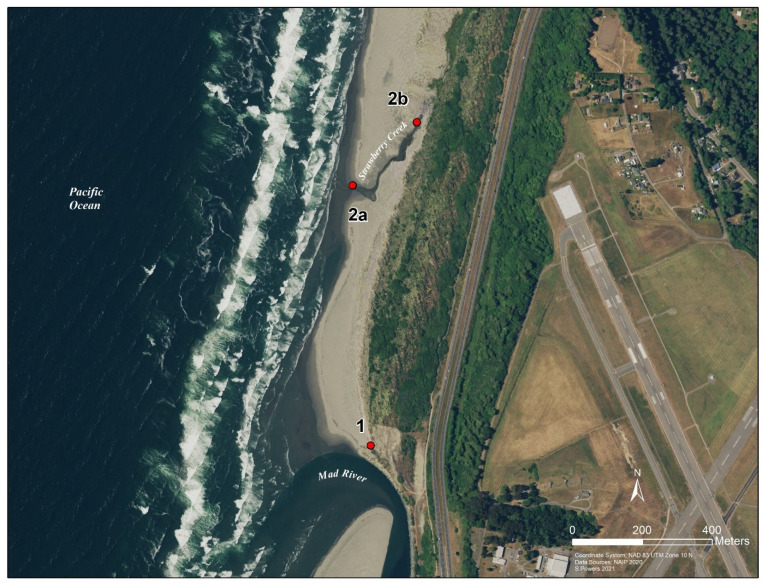
GIS map of the study area showing the three collection sites along Clam Beach. Site 1 at Mad River, site 2a in the mixing zone of Clam Beach and Strawberry Creek, and site 2b upstream Strawberry Creek. County of Humboldt GIS. Per week, typically 17 samples per sampling event. At least 100 mL of water were collected in a DNA-Free Falcon sterile collection container using a sampling pole. All samples were transported to the lab.

For this project there were two collection strategies. The first was an 11-h, single-day collection event at Clam Beach (beach study) and the second was an 8-week-long weekly collection strategy at the Strawberry Creek watershed (watershed study). Samples for the beach study were collected from 7:00 a.m. to 17:30 p.m. at three collection sites along Clam Beach on 6 August 2020 (Figure 2). The number of humans and dogs were directly counted during the collection period, while the number of birds were estimated. Samples were collected every 30 min at sample sites 2a and 2b. Sample site 2a was in the mixing zone of Strawberry Creek and the ocean. Sample site 2b was in Strawberry Creek, which was not influenced by the ocean and is approximately 100 ft. upstream from sample site 2a. For comparison, a third site at Clam Beach near Mad River (site 1) was sampled at low, medium, and high tides only, for a total of 12 samples collected. Replicate samples were taken at low (8:21 a.m.), medium, and high (15:02 p.m.) tides. Samples for the watershed study were taken from 22 May 2020 to 10 July 2020, collected at 13 locations along Clam Beach, Strawberry Creek, Patrick Creek, and Rose Creek (Figure 1). Sample site 2b was excluded in the watershed study. Samples were collected over an 8-week period with samples taken once.

### 2.3. FIB Analysis

Samples were analyzed by defined substrate culture methods for total coliform (TC), EC, and ENT bacteria. TC and EC bacteria were enumerated with IDEXX Colilert−18 or Colilert. Marine samples were analyzed using IDEXX Colilert, whereas freshwater samples were analyzed using IDEXX Colilert−18. ENT bacteria were enumerated with IDEXX Enterolert. Samples were diluted 1/10 in sterile DI water. Results were calculated and reported as most probable number (MPN) per 100 mL of water with a limit of detection <10 MPN.

### 2.4. MST Sample Preparation

Samples containing 100 mL of water were vacuum filtered through a 0.2-µm-pore-size Supor membrane filter (Pall Corporation, Ann Arbor, MI, USA). Filters were placed in microcentrifuge tubes with 0.5-mL glass beads (Sigma# G-1277, 212–300 um), immediately frozen, and stored at −70 °C until DNA extraction. A 0.2 µg/mL of salmon DNA (Sigma Aldrich, St. Louis, MO, USA) in AE buffer (Qiagen, Germantown, MD, USA) was added to each sample and bead beat using a Biospec mini bead beater-16 for 60 s, followed by purification using the MagMAX™ Viral/Pathogen II Nucleic Acid Isolation Kit. Nucleic acid isolation was performed via a newly validated automated process using the KingFisher™ Flex Purification System. All samples were eluted at 100 µL and stored at −70 °C until qPCR analysis. A method blank using sterile PBS was processed exactly as all samples and considered a negative extraction control. Calibration samples with known quantities of *Bacteroides thetaiotaomicron* (ATCC #29741) were also subjected to all of the steps in the protocol and used as a positive extraction control [21].

### 2.5. QPCR Analysis of MST Markers

All environmental samples extracted were tested for MST markers that identify human, ruminant, canine, and bird fecal sources using qPCR. The following primer and probe targets were used: human-associated *Bacteroides* (HF183), ruminant-associated *Bacteroides* (Rum2Bac), canine-associated *Bacteroides* (DogBact), and bird-associated *Catellicoccus marimammalium* (Lee Seagull) [21,22]. For all qPCR analysis, samples were run in duplicate and quantities were determined by comparison to a standard curve. The standard curve was constructed from plasmid DNA containing the target sequence at 10-fold dilutions ranging in concentration from 10^6^ to 10^1^ gene copies per reaction and tested in triplicate for HF183, Rum2Bac, DogBact, and Lee Seagull. In addition, negative controls (no template added) tested in duplicate were added to each qPCR plate run. Gene copy quantities were calculated as the mean concentration of duplicate reactions and reported as copies per 100 mL of water. All assays were carried out in MicroAmp Optical 96-well reaction plates with MicroAmp plate sealers (Applied Biosystems, Foster City, CA, USA). All qPCR assays were performed using an ABI 7500 FAST Dx Real-Time PCR Instrument (Thermo Fisher Scientific, Waltham, MA, USA). PCR data were analyzed using ABI’s Sequence Detection software (version 1.4.1). All qPCR assays were carried out according to Method B and SCCWRP Manual guidelines with minor modifications, and a summary is provided in the supplemental Table S3 [21,22]. Standard curves and limit of detection for all assays were analyzed according to published MIQE guidelines [23].

### 2.6. Statistical Methods

Statistical analyses of FIB and animal-associated MST marker data including descriptive statistics, Spearman rank correlations, and Mann–Whitney U tests were performed using IBM SPSS Statistics software version 25 (International Business Machines, New York, NY, USA). General descriptive statistics include the mean, median, standard deviation, coefficient of variation, and 90th percentile. Censored FIB concentration measurements were estimated and imputed using the regression on order statistics (ROS) method [24]. FIB and MST bird marker that were below limit of quantification (BLOQ) were estimated using ROS for statistical comparison only. A series of Spearman rank correlations were conducted in order to determine if there was an association between FIB and MST markers. A non-parametric test was chosen because the data did not meet the distribution assumptions required for the parametric Pearson’s correlation test. A Mann–Whitney U test was used to test for differences between FIB concentrations at Clam Beach near Mad River (site 1) and Clam Beach near Strawberry Creek (sites 2a and 2b). For statistical analysis and to estimate influence of sunlight, sample collection time was grouped by early morning, morning, or afternoon. Early morning was defined as samples collected between 7:00 a.m. and 8:15 a.m. and was intended to represent bacteria levels at night. Morning was defined as samples collected between 8:30 a.m. and 11:30 a.m. Samples collected the rest of the day were grouped as afternoon.

### 2.7. Regulatory Standards Evaluated

FIB concentrations of collected surface water samples were also assessed against relevant regulatory standards. This study used the California Department of Health Services’ regulations [19] for public beaches and ocean water-contact sports areas (Health and Safety Code 5115880; Assembly Bill 411, Statutes of 1997, Chapter 765). Based on a single daily sample, the concentration of FIB in water from each sampling station at a public beach or public water-contact sports area shall not exceed:

(1) 10,000 total coliform bacteria per 100 milliliters; or
(2) 400 fecal coliform bacteria per 100 milliliters; or
(3) 104 enterococcus bacteria per 100 milliliters.

These criteria represent a human illness risk of 36 gastrointestinal illnesses per 1000 recreators. Illness was defined as symptoms of diarrhea, stomachache, or nausea with or without the occurrence of fever and the definition is referred to as NGI [25]. The acceptable risk of illness from recreational contact with surface waters was an early policy decision by the U.S. EPA (USEPA 1976). This level of acceptable illness risk has been used for all subsequent FIB standards established by USEPA and the State of California. These standards also include criteria based on distributional characteristics of FIB data. This study only evaluated criteria based on the daily SSM concentrations of FIB and did not evaluate distributional criteria due to inadequate number of samples collected.

Additional criteria for two MST markers have also been developed using a quantitative microbial risk assessment approach. Boehm et al., in 2015, determined the median concentration of the human-associated fecal marker HF183 to be approximately 4200 gene copies for 30 illnesses per 1000 recreators. Recently, the water quality threshold for human illness at 32 illnesses per 1000 recreators for the HF183 marker was redefined to be between 1–525 copies per 100 mL of water by taking into account temperature-specific organism decay and co-occurring gull fecal contamination [7]. Brown et al., in 2017, also used the 30 illnesses per 1000 recreators benchmark to determine the bird-associated fecal marker CAT median concentration of 4 × 10^6^ gene copies equivalent illness risk. This study evaluates the FIB criteria illness risk of 36 illnesses per 1000 recreators to be essentially equivalent to the 30 illnesses per 1000 recreators illness risk from measured MST markers, allowing the comparison between FIB and MST marker concentrations based on illness risk.

## 3. Results

What follows is a description of the results of the study divided into two sections. The first is the 11-h beach study at Clam Beach and the second is the 8-week watershed study at the Strawberry Creek watershed.

### 3.1. Beach Study

#### 3.1.1. FIB and MST Analysis

A total of 66 samples were collected, with 12 (18%) samples collected at Clam Beach near Mad River (site 1) and 54 (82%) samples collected at Clam Beach near Strawberry Creek (sites 2a and 2b). A total of 66 (100%) samples had detectable TC bacteria, 58 (88%) samples had detectable EC bacteria, and 54 (82%) samples had detectable ENT bacteria (Table S4). Descriptive statistics of FIB concentrations and ROS-adjusted bird marker concentrations of samples collected from all sites combined are shown in Table S5. Clam Beach near Strawberry Creek (sites 2a and 2b) samples collected did not exceeded the SSM TC objective of 10,000 MPN/100 mL. Only one sample exceeded the SSM fecal coliform objective of 400 MPN/100 mL. A total of 27 (50%) samples exceeded the SSM ENT objective of 104 MPN/100 mL. Interestingly, site 1 did not have any exceedances for EC or ENT but did have two samples that would have resulted in resample due to exceeding the SSM TC objective. At all sites combined, a total of 29 (44%) samples collected exceeded at least one FIB SSM objective, which would have resulted in a resample per state regulations for recreational primary contact use.

All method blanks and quality control were within expected range. The human and ruminant marker was not detected, the dog marker was detected in 6/66 (9%), and the bird marker was detected most frequently, in 43/66 (65%) of samples collected (Table S4). A total of 25 dogs and 20 people were counted during the 11-h period. One dead bird near site 2a was observed and it was estimated that greater than 50,000 birds were counted over the entire day. The study area was divided into sectors and directly counted. The total area was estimated from those counts on an hourly basis.

#### 3.1.2. Comparison of Mad River and Strawberry Creek Collection Sites

There was a statistically significant difference between EC density at Strawberry Creek (Md = 38.6, *n* = 54) and Mad River (Md = 10.7, *n* = 12), Mann–Whitney U = 50.5, *p* < 0.001. There was also a statistically significant difference in ENT density at Strawberry Creek (Md = 39.0, *n* = 54) and Mad River (Md = 8.9, *n* = 12), Mann–Whitney U = 29.0, *p* < 0.001. Distributions of the measured concentrations of EC and ENT bacteria collected from Strawberry Creek and Mad River were compared visually using box and whisker plots (Figure S2a,b). This shows that Strawberry Creek had a significantly higher density of EC and ENT than Mad River.

**Figure 3:**
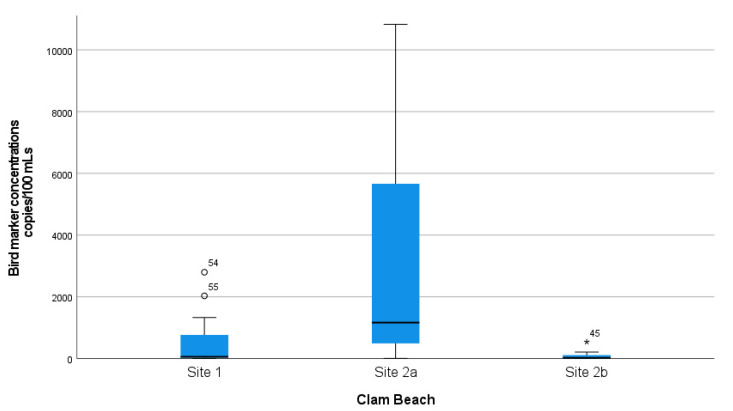
Distribution of bird marker concentrations at all three sampled locations. Mad River (site 1) was a reference site for comparison. Clam Beach near Strawberry Creek (site 2a) was in the mixing zone and considered an ocean sample. Clam Beach near Strawberry Creek (site 2b) was primarily in Strawberry Creek and considered a freshwater sample.

A complete summary of animal–host MST marker concentration measurements in samples collected at Clam Beach near Strawberry Creek is shown in Table 1. Additionally, a summary of MST marker concentration in samples collected at Clam Beach near Mad River is shown in Table 2. The mean distributions of ROS-adjusted bird marker concentrations from 07:30 a.m. to 17:30 p.m. are visually shown in Figure S3, with a single sample spike at noon of 44,058 copies/100 mL. The Kruskal–Wallis test was used to show that there is a statistically significant difference in the distribution of bird marker concentrations between sample collection sites (sites 1, 2a, and 2b), X^2^ (2) = 28.2 and *p* < 0.001. Distributions of the measured concentrations of bird marker concentrations collected at each site were compared visually using box and whisker plots (Figure 3). Furthermore, a Mann–Whitney U test was used to test the hypothesis that the distributions of bird marker concentrations were equal at collection sites 2a (mixing zone) and 2b (freshwater). There was a statistically significant difference in bird marker concentrations at site 2a (Md = 38.6, *n* = 27) and site 2b (Md = 16.4, *n* = 27), Mann–Whitney U = 66.0, *p* <0.001. This demonstrates that the bird marker concentrations and detections were significantly higher in the mixing zone (seawater) when compared to a Strawberry Creek source (freshwater).

#### 3.1.3. Comparison of Collection Time and Time of Day Collected

**Figure 4:**
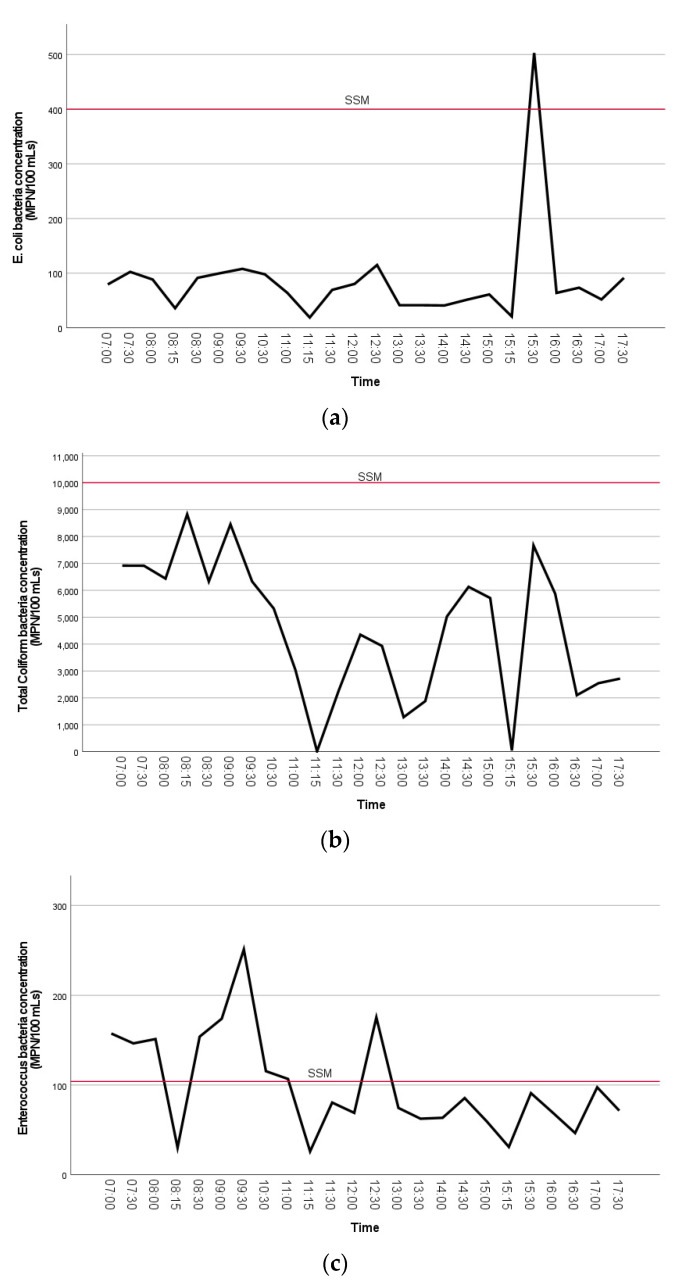
(**a**–**c**). The mean distributions of FIB bacteria concentrations (MPN/100 mL) (**A**. *E. coli*, **B**. total coliform, **C**. enterococci) at Clam Beach near Strawberry Creek (sites 2a and 2b combined) are visually shown in the line plots. The single sample maximum (SSM) threshold is indicated on each plot.

The concentrations of FIB bacteria are visually shown in the line plots below (Figure 4a–c). A Kruskal–Wallis test was used to test the differences of FIB concentrations between sample collection time by group (early morning, morning, afternoon). The Kruskal–Wallis test for comparison of time of day collected showed that there was a statistically significant difference in the distribution of TC bacteria concentrations between groups, X^2^ (2) = 19.1 and *p* < 0.001. Furthermore, there was a statistically significant difference in TC density between early morning (Md = 24.5, *n* = 16) and morning (Md = 12.6, *n* = 19), Mann–Whitney U = 48.5, *p* = 0.001. Distributions of the measured concentrations of TC bacteria collected from each sample time by group were compared visually using box and whisker plots (Figure S4). This shows that early morning concentrations of TC were higher than morning or afternoon samples.

#### 3.1.4. Comparison of Tidal Effects

There was a statistically significant difference in TC density between low tide (Md = 38.9, *n* = 35) and high tide (Md = 27.4, *n* = 31), Mann–Whitney U = 354.5, *p* = 0.016. There was also a statistically significant difference in ENT density between low tide (Md = 38.1, *n* = 35) and high tide (Md = 28.3, *n* = 31), Mann–Whitney U = 383.0, *p* = 0.040. Distributions of the measured concentrations of TC and ENT bacteria collected at low and high tides were compared visually using box and whisker plots (Figure S5a,b). TC and ENT density showed higher densities at low tide when compared to high tide.

Moreover, there was a statistically significant difference in TC density between outgoing tide (Md = 38.8, *n* = 29) and incoming tide (Md = 29.3, *n* = 37), Mann–Whitney U = 383.5, *p* = 0.048. Distributions of the measured concentrations of TC bacteria collected on outgoing tide and incoming tide were compared visually using box and whisker plots (Figure S6). TC density showed higher densities at outgoing tide when compared to incoming tide. We found no significant associations between FIB concentration and MST marker concentrations. However, a statistically significant and relatively strong monotonic association was found between the EC and ENT concentration (Spearman’s rho = 0.691, *p* < 0.001) All other statistically significant associations were relatively weak and are summarized in Table S6. A visual representation of the monotonic association of fecal indicator bacteria EC, TC, and ENT is shown in Figure S7.

### 3.2. Watershed Study

#### 3.2.1. FIB and MST Analysis

A total of 135 (100%) samples had detectable TC bacteria, 121 (89%) samples had detectable EC bacteria, and 122 (90%) samples had detectable ENT bacteria (Table S7). Descriptive statistics of FIB concentrations of samples collected from all sites combined are shown in Table S8. Only collection day 4 and day 7 would have resulted in concentrations high enough to require a re-sample at Clam Beach due to exceeding the SSM. All method blanks and quality control were within expected range. The human marker was detected in 4/135 (3%) samples, the ruminant marker was detected in 41/135 (30%) samples, the dog marker was detected in 7/135 (5%), and the bird marker was detected in 7/135 (5%) of samples collected (Table 3).

Mad River (site 1) was the only sampled site that indicated influence from birds. The footbridge (site 5) and walking path (site 6) were the only sites that had any detectable dog marker in the watershed. Only two locations (sites 8 and 11) had any detectable human marker. A total of 41 samples had detectable ruminant marker, with eight samples quantified (Table S7). Only one site did not show any detectable MST markers (site 9). A summary of key findings at each location for the 8-week study is summarized in Table S9.

#### 3.2.2. Comparison of All 13 Collection Sites and Three Sampled Creeks

A Kruskal–Wallis hypothesis test was used to test the differences of FIB concentrations between sample locations (1–13) because the requirements for using a parametric test were not met and sample sizes within each group were small. The Kruskal–Wallis test was used for comparison of sample collection location (1–13) and showed a statistically significant difference in the distribution of TC and ENT concentrations between sample locations, X^2^ (12) = 48.6 and *p* < 0.001 and X^2^ (12) = 39.0 and *p* < 0.001, respectively. Distributions of the measured concentrations of TC and ENT bacteria concentrations collected at each site were compared visually using box and whisker plots (Figure S8). Sampling sites 2, 3, 4, and 5 showed the highest concentration of TC density. Sample site 8 showed the highest density of ENT.

Mad River (site 1) was the only sampled site that indicated influence from birds, with 4/8 samples detected BLOQ. The footbridge (site 5) and walking path (site 6) were the only sites that had any detectable dog marker, with one detection at site 5 at 4200 copies/100 mL. Only two locations (site 8 and site 11) had any detectable human marker. Site 8 had one sample at 591 copies/100 mL and the pond off of Strawberry Creek had one sample at 629 copies/100 mL. A total of 41 samples had detectable ruminant marker with eight samples quantifiable, ranging from 588 copies/100 mL to 2089 copies/100 mL at 10/13 sampled locations (Table S7). Patrick Creek at Dows Prairie Rd. (site 9) was the only site that did not show any detectable MST markers.

Furthermore, the Kruskal–Wallis test was used for comparison of the three creeks sampled (Strawberry Creek, Patrick Creek, and Rose Creek) and showed that there was a statistically significant difference in the distribution of ENT concentrations between these creeks, X^2^ (2) = 7.2 and *p* = 0.027. Distributions of the measured concentrations of ENT bacteria concentrations collected at each creek were compared visually using box and whisker plots (Figure S8). This showed that Patrick Creek had a higher density of ENT than Strawberry Creek and Rose Creek.

#### 3.2.3. Comparison of Sampling Events and Drainage Areas

The Kruskal–Wallis test was used for comparison of the eight sampling days and showed that there was a statistically significant difference in the distribution of *EC*, TC, and ENT concentrations between groups: X^2^ (7) = 29.9 and *p* < 0.001, X^2^ (7) = 49.2 and *p* < 0.001, and X^2^ (7) = 45.8 and *p* < 0.001, respectively. Distributions of the measured concentrations of FIB concentrations for each sampling event were compared visually using box and whisker plots (Figure S10). Additionally, the Kruskal–Wallis test was used for comparison of the seven drainage areas and showed a statistically significant difference in the distribution of *EC*, TC, and ENT concentrations between drainage areas: X^2^ (6) = 14.0 and *p* = 0.030, X^2^ (6) = 34.0 and *p* < 0.001, and X^2^ (6) = 21.9 and *p* = 0.001, respectively. Distributions of the measured concentrations of FIB concentrations for each drainage area were compared visually using box and whisker plots (Figure S11).

## 4. Discussion

Several approaches were used to help identify the potential source(s) of fecal contamination to better understand Clam Beach near Strawberry Creek. For the pilot project, the 3 years’ worth of weekly sample data indicated that birds were the most frequent source consistently detected throughout the year. As a result, we designed a short-term, targeted study at Clam Beach that showed frequent bird marker detections with no detectable human or ruminant sources. This suggests that failing septic systems or pastured cattle, wild deer, and elk are not likely to be a major contributor to fecal contamination. Nearly half (50%) of the FIB samples collected at Clam Beach near Strawberry Creek during the ocean beach study period exceeded the SSM objectives, which would have prompted posting a beach advisory and subsequent re-testing until levels returned to acceptable levels. This location is meeting the TC objective. However, there were resamples due to exceedances of EC and ENT. Only two (17%) of the samples collected at Clam Beach near Mad River (site 1) would have resulted in a resample, which was due to exceedance of the TC SSM objective. Strawberry Creek had a higher density of EC and ENT bacteria density when compared to Mad River, which could be attributed to the size of the streamflow. It should be noted that during the watershed study, no sample exceeded any FIB SSM objective at site 1. In addition, site 2a had only two (17%) exceedances due to ENT, which would have resulted in a resample. Mad River’s flow is significantly larger than Strawberry Creek, which is perhaps flushing the bacteria levels further out into the ocean.

In the beach study, the second most-abundant MST marker detected was the dog marker (9%) but none of the detections were quantifiable. Throughout the day several dogs were observed, which was consistent with the low level of dog MST marker detections, none of which appeared to pose any human health risk since all six samples detected were BLOQ. The most abundant MST marker detected was the bird marker (65%), with a little less than half of the samples in the quantifiable range. In addition, no sample had co-occurring bird and human marker, which would have lowered the potential risk threshold [7]. Despite the high frequency of bird marker detections, no sample exceeded the QMRA derived criteria of 4 × 10^6^ copies per 100 mL, which correlates to the USEPA benchmark of 30 GI illnesses per 1000 swimmers [5,26]. Despite the strong presence of bird marker there was no association with any of the FIB, which is consistent with previous studies [27]. The day of this study, both Strawberry Creek and Mad River beaches showed striking visible influence from birds.

Environmental factors play an important role in FIB concentration and can influence the density throughout the entire day, which was shown in this study by the fluctuating detected FIB concentrations [4]. The laboratory data produced were consistent with the literature on known spatial and tidal patterns of fecal contamination [28,29]. The detection of significantly higher TC density in the early morning was consistent with the known effects of sunlight on FIB concentration in water [30]. Tides also play a critical role in FIB levels, making it extremely challenging to evaluate true health risk. As with many other studies, TC and ENT densities were higher at low tide when compared to high tide [31]. Additionally, TC density was higher during outgoing tide when compared to incoming tide, highlighting the complex nature of beach sampling and monitoring. Birds influence fecal contamination at Clam Beach, but sample location plays an important factor in bird marker detections. The samples taken in the mixing zone (site 2a) had much higher bird marker density when compared to the sample taken in Strawberry Creek (site 2b), which was freshwater. While sampling, thousands of birds were observed bathing between the two sample locations: upstream of 2a and downstream from 2b. In addition, there was one dead bird found between the sampling locations, which may have contributed to the findings of this study. It also highlights the importance of taking a strategic approach to sampling, especially if health risk is substantial or regulatory decisions are being based on these data [32].

The watershed study was used to determine if the Strawberry Creek watershed was contributing directly to the increased FIB levels found at Clam Beach. Potential sources varied by sample location. The Mad River site 1 was the only sample location that showed any influence from birds, which is consistent with the Beach Study. The Strawberry Creek watershed did not show any influence from birds. The only site that showed influence from dogs was the footbridge (site 5). Interestingly, one sample collected at this site exceeded all three FIB markers and had 4200 copies/100 mL of the dog marker detected. There were two quantifiable human detections: one at site 8 (Patrick Creek) and one at site 11 (the pond). Both detections were at the water quality threshold of 525 copies/100 mL, indicating potential health risk [7]. However, these two sites are distant from Clam Beach and did not appear to affect this location. Furthermore, Patrick Creek showed higher ENT when compared to the other creeks, suggesting further study of this creek is necessary. The most important finding was the consistent ruminant detections throughout the Strawberry Creek watershed and the Clam Beach near Strawberry Creek site 2a. Ruminants appear to be more influential than originally thought, which is not surprising due to the land use in this region. MST methods are still evolving. In this study, we were successful in automating the extraction process, which was key in being able to integrate these methods in a public health laboratory setting. Moreover, future research could focus on improving the methodology, making it easier to adapt in a variety of laboratory settings. First, bridging extraction and PCR platforms to these methods will make adoption more palatable if labs can add these methods without significant capital investment for equipment. Using familiar equipment and similar workflows is attractive to labs considering adding to their testing menu. Second, improving the extraction control by blending the human, ruminant, dog, and bird marker into one calibrator sample with known quantities to serve as a single extraction control is needed. It would improve quality control by minimizing sample controls while lowering cost. Lastly, advancing these molecular assays into a multiplex option would greatly improve these methods and cut the work and time by 25%. Results in real time are needed to make this a practical solution to water quality management. The way the assays are designed now, multiple thermocyclers are needed to acquire data that are actionable. In the future, the use of MST multiplex assays with similar sensitivity would significantly reduce both reagent costs and technical time expenses. This would make it easier for labs to integrate these protocols into existing workflows.

## 5. Conclusions

Clam Beach near Strawberry Creek has been consistently identified for having high FIB detections and has been publicly named as one of the top 10 “bad beaches” in California. Routine sampling often leads to beach postings and warnings to the general public. In addition, these SSM exceedances lead to resamples and follow-up laboratory testing until the levels return to acceptable limits. This approach to beach monitoring can result in the use of more resources and often unclear answers to actual human health risk. With MST added to the current analysis we can provide a better assessment of human health risk and potential sources of fecal contamination. However, they often do not correlate to FIB levels. Our data indicated that failing septic systems are not likely to be the source of fecal contamination and demonstrated that the high concentrations of bird fecal matter at Clam Beach is likely due to a natural population of nearshore birds regularly observed along Clam Beach.

MST methods were used, which identified birds as a potential vector for human pathogens, potentially increasing human risk for illness [11,15,16,33,34]. This is important because the median risk of illness using HF183 (human fecal marker) is three orders of magnitude less than similar CAT (bird fecal marker) concentrations [15,16,26,34]. Understanding the source of FIB exceedances is invaluable information for recreational water managers to more accurately evaluate the human health risks. In this study, we showed that the FIB exceedances were most likely due to bird sources. Therefore, the probability of illness is lower than if the source were human. Since the potential source was determined to be bird, it is reasonable to conclude that risks to human health are within the regulatory illness risk benchmark of approximately 30 illnesses per 1000 recreators [5]. Clam Beach is frequented by birds, which adds to the allure of its location and should be celebrated not feared. Perhaps in the future, the grading methodology used to create the “Beach Bummer List” could take into account MST data when evaluating human health risk.

## Figures and Tables

**Table 1:** Summary of animal–host microbial source tracking marker concentration measurements in samples collected at Clam Beach near Strawberry Creek (sites 2a and 2b).

Sample Results	Number and Percent of Animal-Host Markers			
	Human	Ruminant	Dog	Bird
Not Detected	54/54 (100%)	54/54 (100%)	48/54 (89%)	21/54 (39%)
BLOQ *^a^*	0/54 (0%)	0/54 (0%)	6/54 (11%)	6/54 (11%)
Quantified	0/54 (0%)	0/54 (0%)	0/54 (0%)	27/54 (50%)

*^a^* BLOQ = below limit of quantification.

**Table 2:** Summary of animal–host microbial source tracking marker concentration measurements in samples collected at Clam Beach near Mad River (site 1).

Sample Results	Number and Percent of Animal-Host Markers			
	Human	Ruminant	Dog	Bird
Not Detected	12/12 (100%)	12/12 (100%)	12/12 (100%)	2/12 (17%)
BLOQ *^a^*	0/12 (0%)	0/12 (0%)	0/12 (0%)	6/12 (50%)
Quantified	0/12 (0%)	0/12 (0%)	0/12 (0%)	4/12 (33%)

*^a^* BLOQ = below limit of quantification.

**Table 3:** Summary of animal–host microbial source tracking marker concentration measurements in samples collected at all sites combined for the 8-week study.

Sample Results	Number and Percent of Animal-Host Markers			
	Human	Ruminant	Dog	Bird
Not Detected	131/135 (97%)	94/135 (70%)	128/135 (95%)	128/135 (95%)
BLOQ *^a^*	2/135 (1.5%)	32/135 (24%)	6/135 (4%)	7/135 (5%)
Quantified	2/135 (1.5%)	9/135 (6%)	1/135 (1%)	0/135 (0%)

*^a^* BLOQ = below limit of quantification.

## Data Availability

Data is contained within the article or supplementary material.

## Supplementary Materials

The following are available online at https://www.mdpi.com/article/10.3390/ijerph18136901/s1. The supporting information is submitted as an attachment and referenced throughout the manuscript. Figure S1: Primary land use by area of Strawberry Creek watershed. County of Humboldt GIS. Figure S2a,b: Distribution of fecal indicator bacteria *E. coli* and enterococci concentrations in samples collected at Clam Beach near Strawberry Creek and Clam Beach near Mad River for the 11-h study. The single sample maximum (SSM) threshold is indicated on the plot. The boxes represent the interquartile range distribution around the median and the whiskers represent the 10th and 90th percentiles. Figure S3: The mean distributions of bird marker concentrations from 07:30 a.m. to 17:30 p.m. are shown. Figure S4: Distribution of TC concentrations in samples collected at early morning (7:00 a.m.–8:15 a.m.), morning (8:30 a.m.–11:30 a.m.), and afternoon (12:00 p.m.–17:30 p.m.). The single sample maximum (SSM) threshold is indicated on the plot, Figure S5a,b: Distribution of total coliform and enterococci concentrations at low and high tide at all locations sampled. The single sample maximum (SSM) threshold is indicated on the plot. Figure S6: Distribution of total coliform bacteria concentrations at incoming and outgoing tides at all locations sampled. The single sample maximum (SSM) threshold is indicated on the plot. Figure S7: Scatter plot of fecal indicator bacteria E. coli, total coliform, and enterococci. Figure S8: Distribution of total coliform and enterococci bacteria concentrations at each location sampled. The single sample maximum (SSM) threshold and SHELL bacteria objective are indicated. Figure S9: Distribution of enterococci bacteria concentrations at each creek sampled. Figure S10: Distribution of FIB concentrations at each sampling event. Figure S11: Distribution of FIB bacteria concentrations at each drainage area. The single sample maximum (SSM) threshold and SHELL bacteria objective are indicated. Table S1: Summary of animal–host microbial source tracking marker concentration measurements in samples collected at Clam Beach near Strawberry Creek during the dry weather beach monitoring season from August 2015 to April 2018. Table S2: Summary of the sanitation survey conducted for all collection sites selected for this project. Table S3: Description of qPCR parameters used for this study. Table S4: Laboratory results for animal–host microbial source tracking marker concentrations and fecal indicator bacteria concentrations for the 11-h study. Table S5: Descriptive statistics of fecal indicator bacteria and the ROS (regression on order statistics)-adjusted bird microbial source tracking marker in samples collected for the 11-hr study at all sites combined. Table S6: Summary of Spearman rank correlations conducted between fecal indicator bacteria in samples collected at Clam Beach at Strawberry Creek and Clam Beach near Mad River. Table S7: Laboratory results for animal–host microbial source tracking marker concentrations and fecal indicator bacteria concentrations. Table S8: Descriptive statistics of fecal indicator bacteria in all samples collected for the 8-week study at all sites combined. Table S9: A summary of each location, sample site characteristics, and key findings of the 8-week study. Exceedance indicates when the sample exceeded the single sample maximum limits. Table S10: Summary of master mix components and performance characteristics of each MST assay that includes limit of detection (LOD), limit of quantification (LOQ), and LOQ per 100 mL of water filtered.
