# Supplementary material for: Microbial Source Tracking Approach to Investigate Fecal Waste at the Strawberry Creek Watershed and Clam Beach, California, USA
Source: Int J Environ Res Public Health. 2021 Jun 27;18(13):6901. doi: 10.3390/ijerph18136901 (PMC8297226; doi:10.3390/ijerph18136901)
Supplement: Supplementary file 1 [file ijerph-18-06901-s001.zip › ijerph-1251997-supplementary.pdf]

International Journal of
Environmental Research and Public Health

MDPI

Supplementary Material

Microbial Source Tracking Approach to Investigate Fecal Waste at the Strawberry Creek Watershed and Clam Beach, California, USA

Jeremy A. Corrigan [1,2,*], Steven R. Butkus [3], Michael E. Ferris [1], Jill C. Roberts [2]

[1] Humboldt County Public Health Laboratory, County of Humboldt, 529 I Street, Eureka, CA 95501, USA; Jeremy A. Corrigan: jcorrigan@co.humboldt.ca.us; Michael E. Ferris: micromikepetaluma@gmail.com
[2] College of Public Health, University of South Florida, 13201 Bruce B. Downs Blvd, MDC 56, Tampa, FL 33612, USA; jcrobert@usf.edu
[3] State of California, North Coast Regional Water Quality Control Board, 5550 Skylane Blvd., Santa Rosa, CA, 95403, USA; stevebutkus@yahoo.com
* Correspondence: jcorrigan@co.humboldt.ca.us; Tel.: +1-(707)-268-2178

METHODS

Sample Collection. All samples were transported and stored in coolers with ice packs and were received by the lab and processed within 6 hours of collection. A runner was used to pick up samples from the collection site in batches and drive them to the laboratory for processing and analysis.

qPCR Conditions. The qPCR components of each assay are described in Table S5. All assays consist of Taqman Environmental Mastermix 2.0 (Applied Biosystems, Foster City, CA), target specific primers and probes, bovine serum albumin (BSA) (Fisher Scientific, Waltham MA), and nuclease free water (Table S4). The qPCR thermocycler conditions are as follows: 2 min at 50°C, 10 min at 95°C, 40 cycles of 15 sec at 95°C, 1 min at 60°C, ROX dye is on and Ct is set to 0.08. A summary of the performance characteristics is also listed in Table S10.

Interpretation and Analysis of qPCR. Limits of quantification (LOQ) are based on the standard curve and defined as the lowest standard concentration that consistently amplifies within the ROQ. The lower limit of quantification is defined as the LOQ, and in all assays used in this project is determined to be 10 copies per qPCR reaction. The limit of detection (LOD) of the qPCR assays are defined as the theoretical lower limit of detection which is 1 copy per qPCR reaction and results below the LOD are characterized as non-detectable (ND). qPCR results below the LOQ (10 copies per qPCR reaction) but above the LOD (1 copy per qPCR reaction) are characterized as detected below limit of quantification (BLOQ). If both replicates failed to amplify, the result was deemed ND. If one replicate failed to amplify, the result was determined to be ND. If the mean of both replicates amplified fell below the LOD, it was determined to be ND. Copies of target sequence per 100 mLs was determined by taking the copies detected in the PCR reaction, dividing by the volume (2 or 5 ul) of purified nucleic acid eluate used in the reaction, multiplying by the total volume (100 ul) of eluate obtained in the purification of the processed lysate, dividing by 100 mL (target volume of water to be filtered), multiplying by the ratio of the *target* volume to be filtered (100 mL) to the *actual* volume of sample water filtered. In short, multiply the resulting copy number by 50 for the human, ruminant and dog assays, and multiply by 20 for the bird assay if the mean copy number is ≥10 copies per qPCR reaction.

SUPPLEMENTAL FIGURES

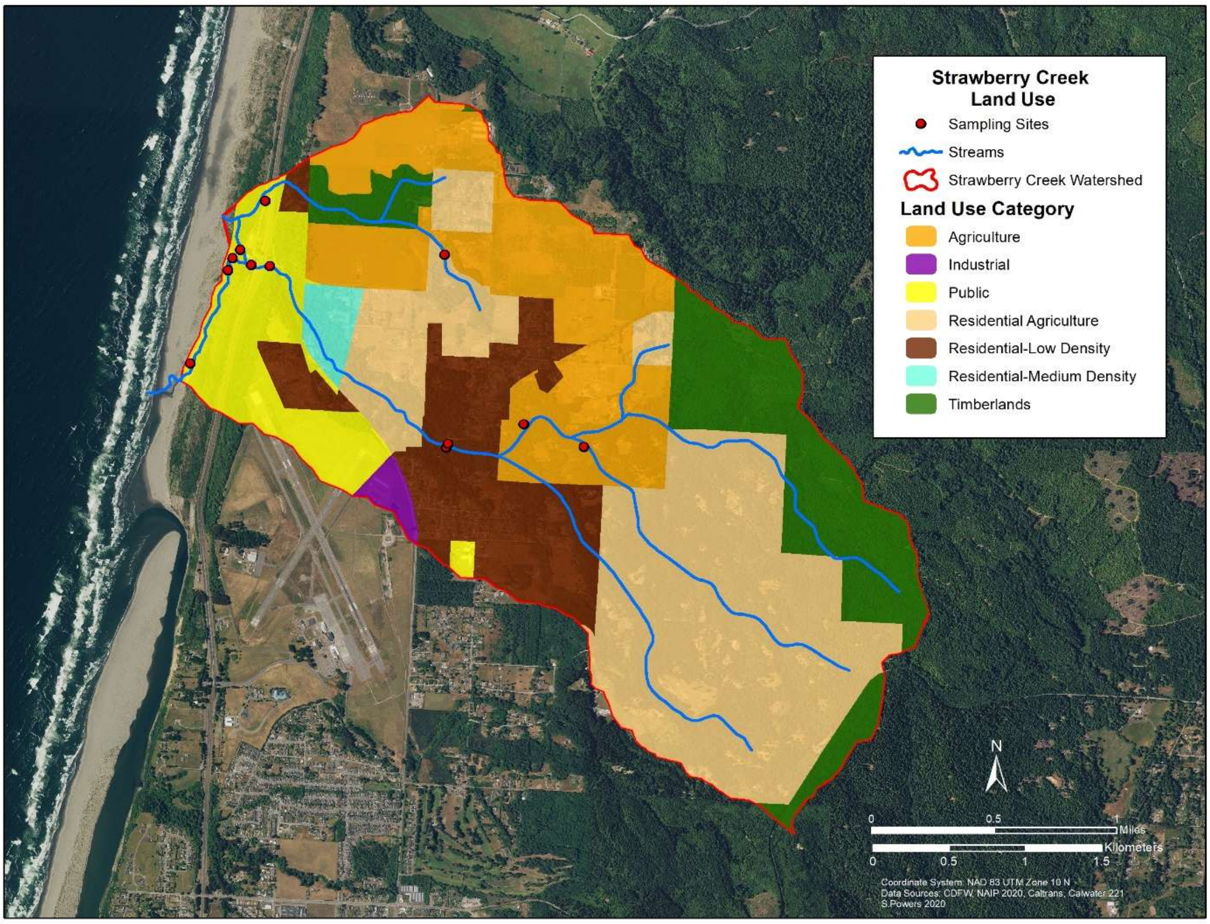

Figure S1. Primary land use by area of Strawberry Creek watershed. County of Humboldt GIS.

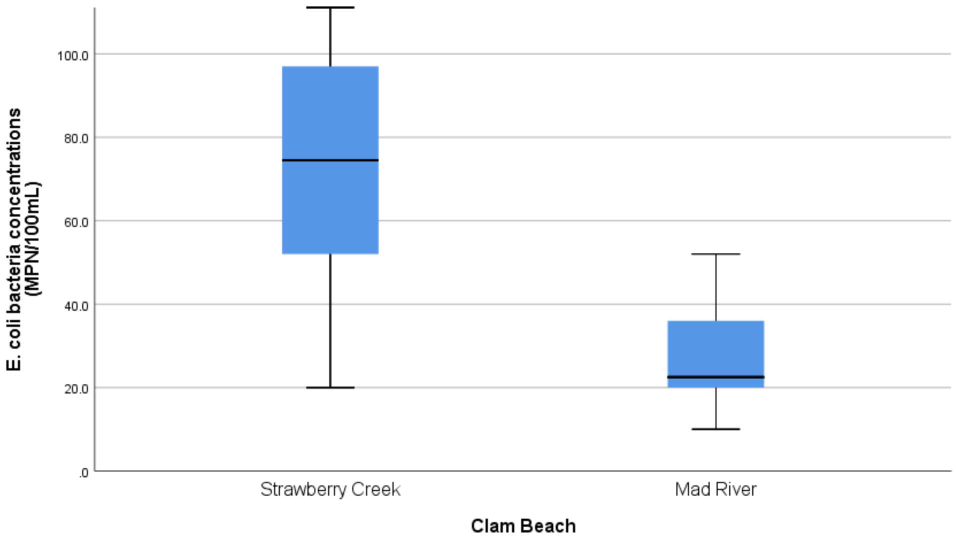

a

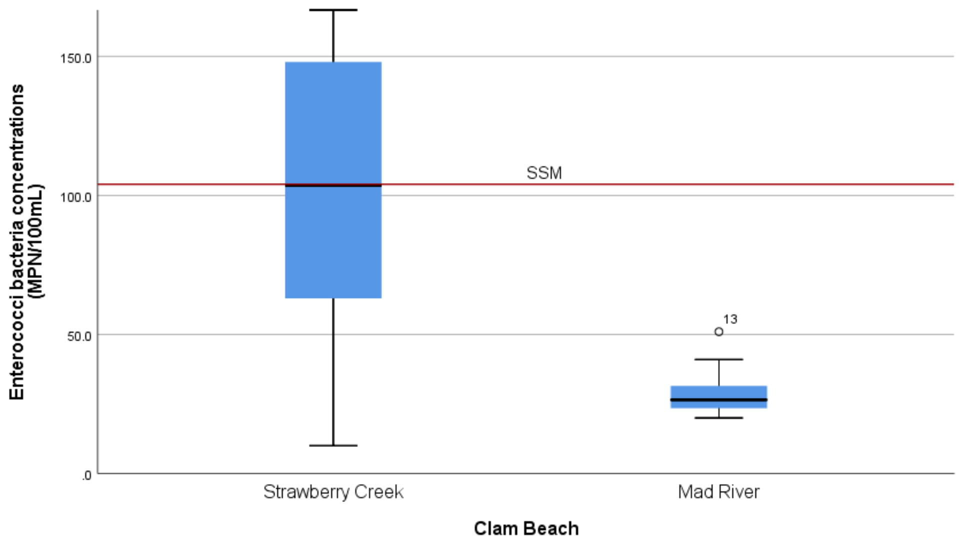

b

Figure S2. a-b. Distribution of fecal indicator bacteria *E. coli* and enterococci concentrations in samples collected at Clam Beach near Strawberry Creek and Clam Beach near Mad River for the 11-hour study. The single sample maximum (SSM) threshold is indicated on the plot. The boxes represent the interquartile range distribution around the median and the whiskers represent the 10th and 90th percentiles.

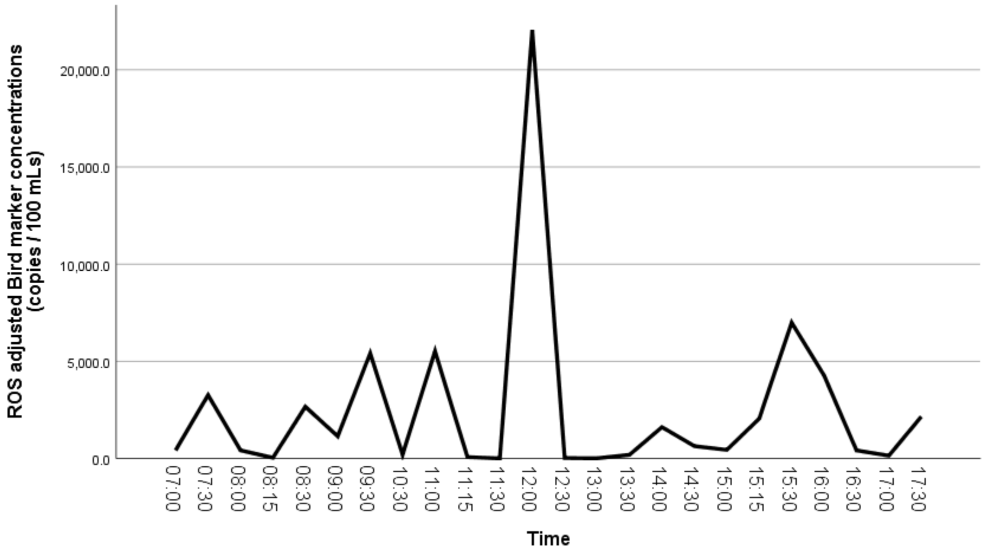

Figure S3. The mean distributions of bird marker concentrations from 07:30 to 17:30 are shown.

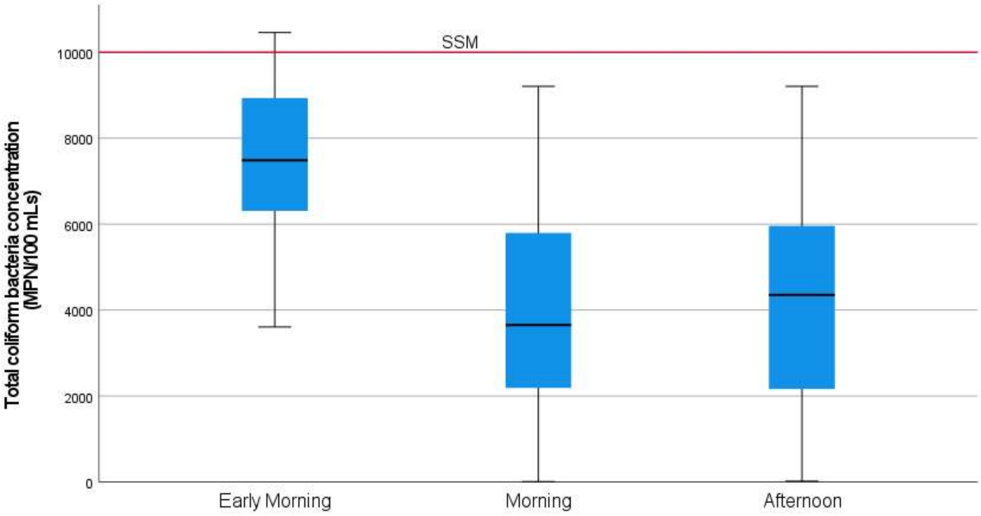

Figure S4. Distribution of total coliform concentrations in samples collected at early morning (7:00 am – 8:15 am), morning (8:30 am – 11:30 am) and afternoon (12:00 pm – 17:30 pm). The single sample maximum (SSM) threshold is indicated on the plot.

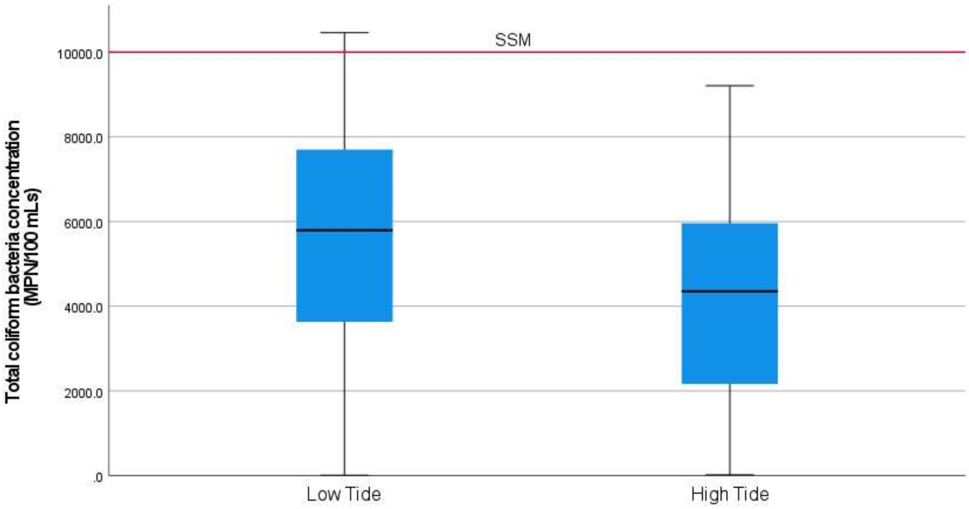

(a)

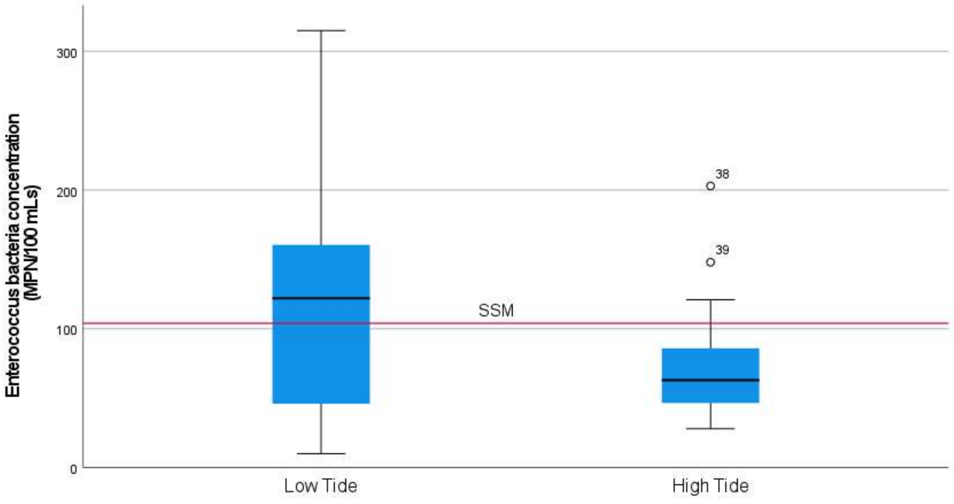

(b)

Figure S5. a-b. Distribution of total coliform and enterococci concentrations at low and high tide at all locations sampled. The single sample maximum (SSM) threshold is indicated on the plot.

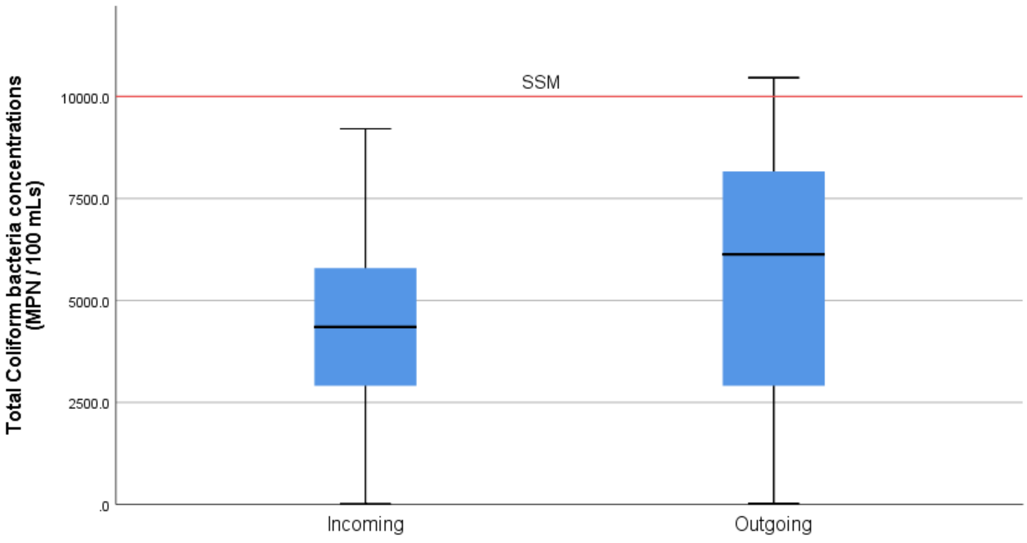

Figure S6. Distribution of total coliform bacteria concentrations at incoming and outgoing tide at all locations sampled. The single sample maximum (SSM) threshold is indicated on the plot.

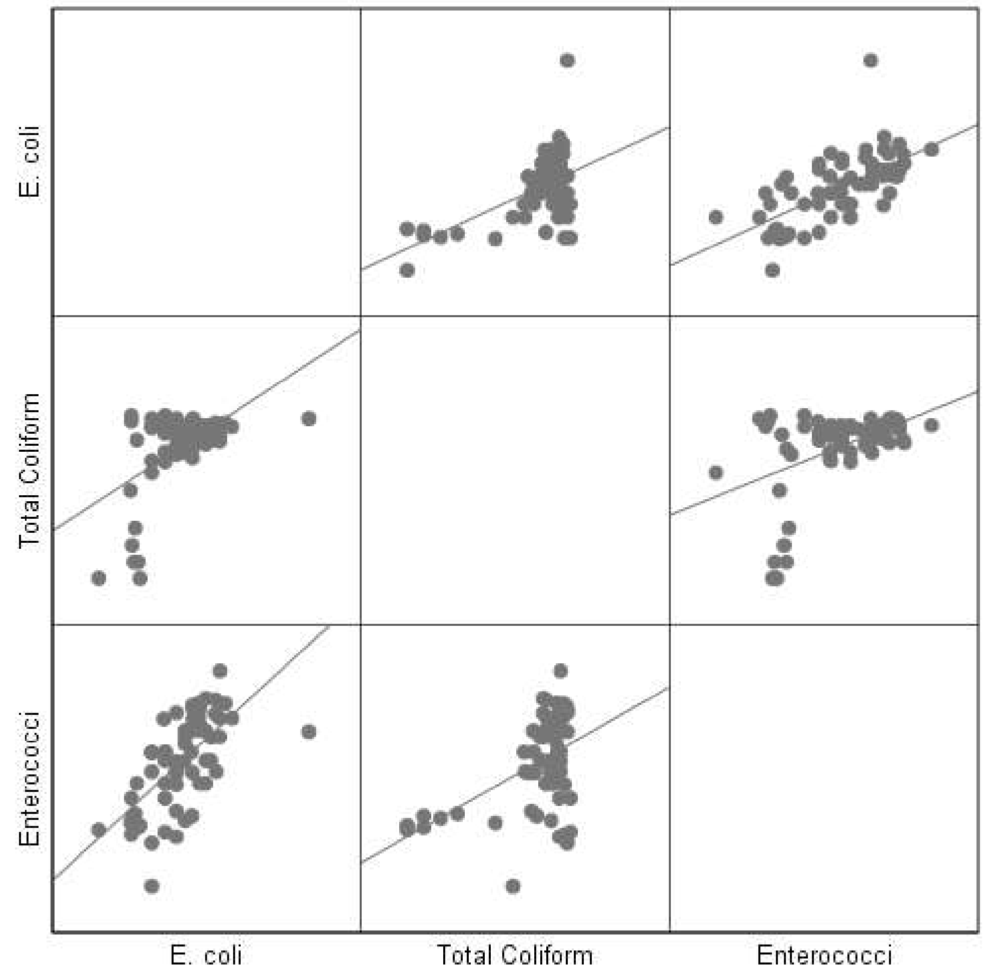

Figure S7. Scatter plot of fecal indicator bacteria *E. coli*, total coliform and enterococci.

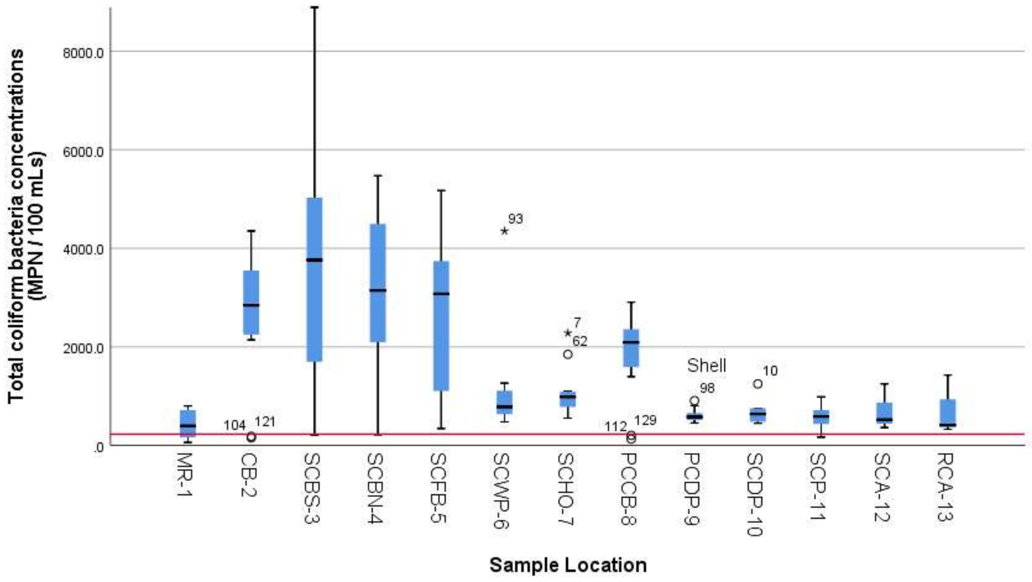

(a)

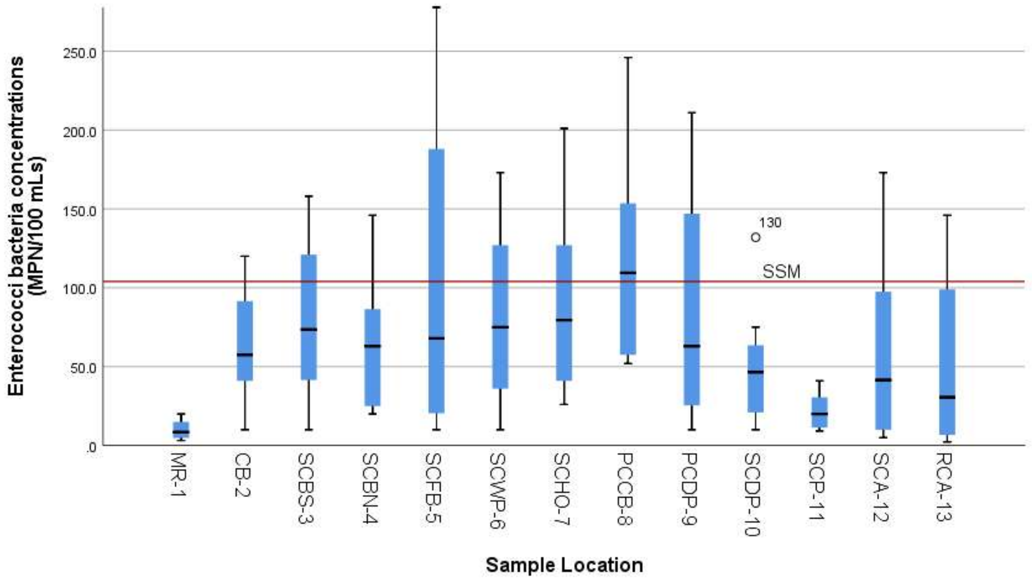

(b)

Figure S8. Distribution of total coliform and enterococci bacteria concentrations at each location sampled. The single sample maximum (SSM) threshold and SHELL bacteria objective is indicated.

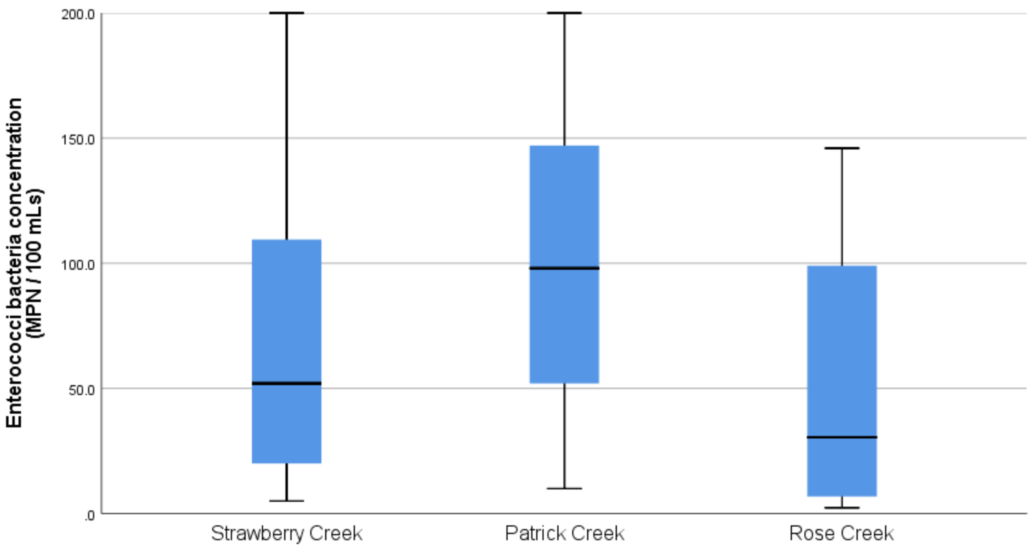

Figure S9. Distribution of enterococci bacteria concentrations at each creek sampled.

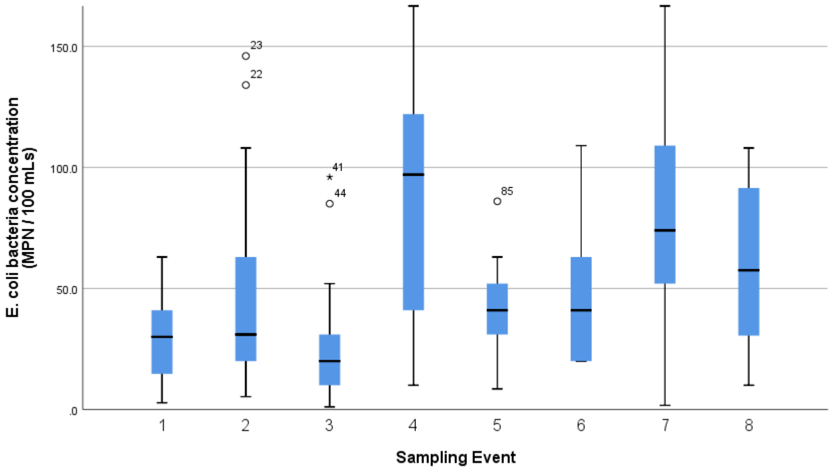

(a)

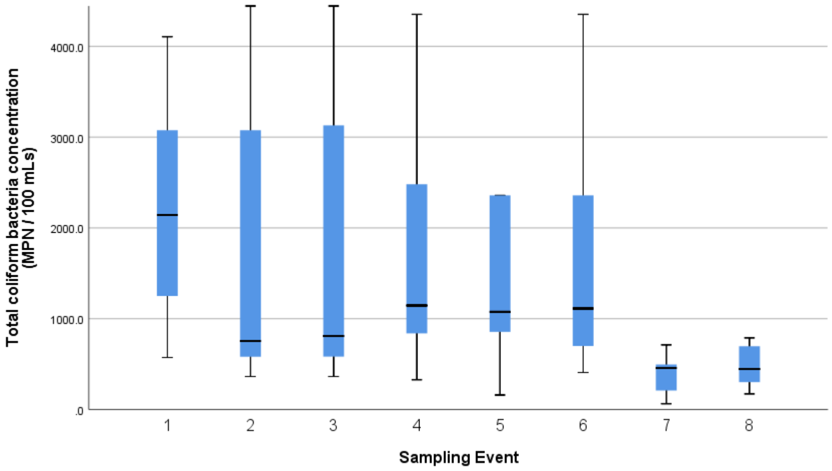

(b)

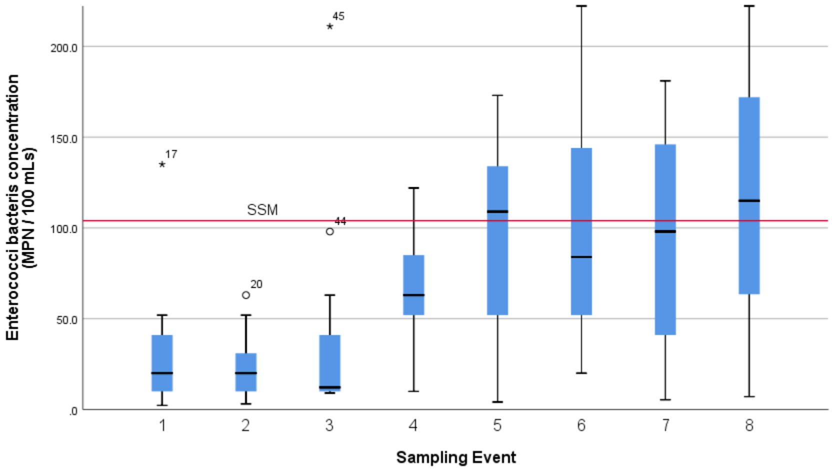

(c)

Figure S10. Distribution of FIB concentrations at each sampling event. .

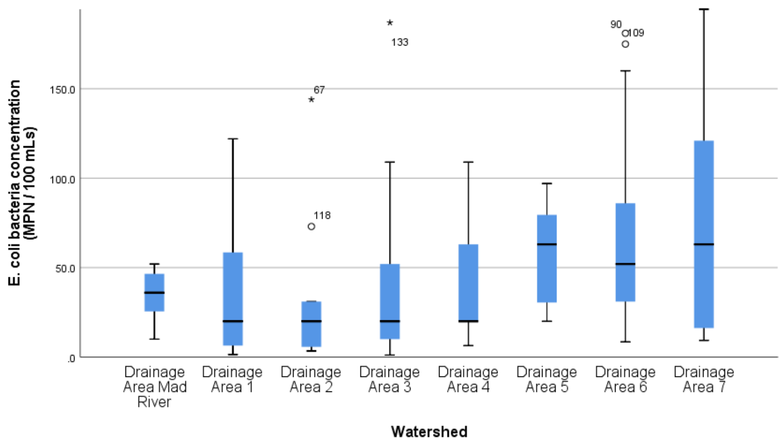

(a)

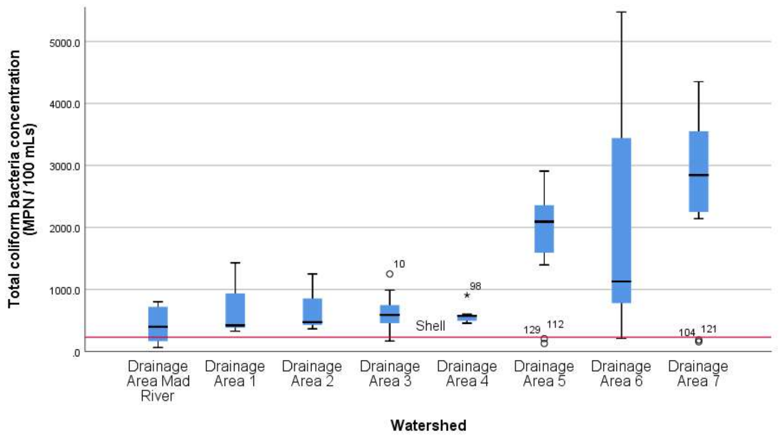

(b)

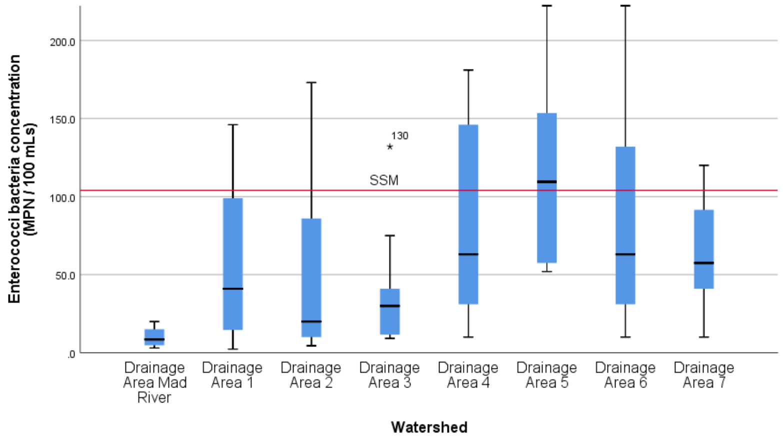

(c)

Figure S11. Distribution of FIB bacteria concentrations at each drainage area. The single sample maximum (SSM) threshold and SHELL bacteria objective are indicated.

SUPPLEMENTAL TABLES

Table S1. Summary of animal-host microbial source tracking marker concentration measurements in samples collected at Clam Beach near Strawberry Creek during the dry weather beach monitoring season from August 2015 to April 2018.

Sample Results	Number and Percent of Animal-Host Markers			
	Human	Ruminant	Dog	Bird
Not Detected	95/103 (92%)	85/103 (82%)	58/103 (56%)	29/103 (28%)
BLOQ[a]	7/103 (7%)	13/103 (13%)	38/103 (37%)	31/103 (30%)
Quantified	1/103 (1%)	5/103 (5%)	7/103 (7%)	43/103 (42%)

[a]BLOQ = Below Limit of Quantification

Table S2. Summary of the sanitation survey conducted for all collection sites selected for this project.

Site ID	Location Description (# of samples)	Significant Characteristics of Study Site and Land Use
Site 1	Mad River (8)	• At mouth of Mad River and Clam Beach<br>• Routine county beach sampling site<br>• Not impaired and used as reference location to CB-2
Site 2a	Clam Beach (12)	• At mouth of Strawberry Creek and Clam Beach<br>• Routine county beach sampling site<br>• Represents drainage area 7 – entire study area, ocean site, public<br>• Impaired and location of interest
Site 3	Strawberry Creek – Beach South (8)	• Along Strawberry Creek, most south sample – freshwater<br>• Represents drainage area 6 – Strawberry Creek, public land use
Site 4	Strawberry Creek – Beach North (12)	• Along Strawberry Creek, slightly north of SITE 3 – freshwater<br>• Represents drainage area 6 – Strawberry Creek, public land use
Site 5	Strawberry Creek -Foot-bridge (12)	• Foot bridge and main access point to beach, public land use<br>• Most human and dog activity concentrated in one spot<br>• Represents drainage area 6 – Patrick Creek & Strawberry Creek
Site 6	Strawberry Creek – Walking Path (12)	• Walking path over Strawberry Creek – frequently used by bikers, walkers, runners, horses and dogs<br>• Not influenced by Patrick Creek watershed<br>• Represents drainage area 6 – Strawberry Creek only<br>• Public and medium density residential land use
Site 7	Strawberry Creek – Highway Offramp (12)	• Not influenced by Patrick Creek watershed or public access<br>• Represents drainage area 6 – Strawberry Creek only<br>• Public and medium density residential land use
Site 8	Patrick Creek – near Clam Beach (12)	• Patrick Creek mouth before it reached Strawberry Creek<br>• Represents drainage area 5 and Patrick Creek watershed<br>• Public, agriculture and low-density residential land use
Site 9	Patrick Creek – Dows Prairie Rd. (12)	• Upstream Patrick Creek – primarily agriculture<br>• Represents drainage area 4 and Patrick Creek watershed<br>• Agriculture and medium density residential land use
Site 10	Strawberry Creek – Dows Prairie Rd. & Arthur Lane (8)	• About 1,600 meters upstream Strawberry Creek<br>• Represents drainage area 3<br>• Duke Creek and Strawberry Creek watersheds<br>• Medium density residential land use
Site 11	Strawberry Creek Pond – Dows Prairie Rd. & Arthur Lane (12)	• Pond adjacent to Strawberry Creek, 100 ft. from SITE 10<br>• Represents drainage area 3<br>• Duke Creek & Strawberry Creek watersheds<br>• Medium density residential land use
Site 12	Strawberry Creek – Arthur Lane (8)	• About 2,100 meters upstream Strawberry Creek<br>• Represents drainage area 2 and Strawberry Creek watershed<br>• Agriculture and timberland land use
Site 13	Rose Creek – Arthur Lane (8)	• 2,500 meters upstream Strawberry Creek @ mouth of Rose Creek<br>• Represents drainage area 1 – Rose Creek watershed<br>• Agriculture land use

Table S3. Description of qPCR Parameters Used for this Study.

Analytical Step	Critical Parameters to be Reported with Results	Study Parameters
Sample Preparation	Volume of water analyzed and filtration method(s)	100 mL sample filtered with Supor membrane (pore size 0.2um) (Pall Corporation, Ann Arbor, MI)
Sample Preparation	Method used to extract nucleic acids	A 0.2 µg/mL of salmon DNA (Sigma Aldrich, St. Louis, MO) in AE buffer (Qiagen, Germantown, MD) was added to each sample and bead beat using a Biospec mini bead beater-16 for 60 seconds, followed by purification using the MagMAX™ Viral/Pathogen II Nucleic Acid Isolation Kit. Nucleic acid isolation was performed via an automated process using the KingFisher™ Flex Purification System.
Sample Preparation	DNA yield and purity	Samples will not be assessed for purity and DNA yield. Just for inhibition for Sketa and/or IAC.
Detection of MST Target	Instrument used for amplification	ABI 7500 Fast Dx, SDS software version 1.4.1, Applied Biosystems.
Detection of MST Target	Evidence for absence of inhibition	Use of Sketa sample processing and inhibition control, in accordance with EPA Method B. Use of IAC in the HF183 PCR assay, in accordance with the SCCWRP - California Microbial Source Identification Manual.
Detection of MST Target	Definition of positive detection	Detection within the linear dynamic range of the method accompanied by expected results from controls.
Detection of MST Target	Definition of limit of detection (LOD)	The LOD of the qPCR assays are defined as the theoretical lower limit of detection which is 1 copy per qPCR reaction and results below the LOD are characterized as non-detectable (ND)
Detection of MST Target	Definition of limit of quantification (LOQ) and LLOQ	The lower limit of quantification (LLOQ) is defined as the LOQ, and in all assays used in this project is determined to be 10 copies per qPCR reaction.
Detection of MST Target	Definition of below limit of quantification (BLOQ)	qPCR results below the LOQ (10 copies per qPCR reaction) but above the LOD (1 copy per qPCR reaction) are characterized as detected below limit of quantification (BLOQ)
Quantification of MST Target	Calibration curve with slope, y-intercept, r', and efficiency amplification	Y = MX +B; amplification efficiency (E) = (10^(-1/slope))-1
Quantification of MST Target	Reference material used to generate a standard curve	The GenBac assay will use genomic DNA, whereas all species-specific markers Plasmid DNA will be used.
Quantification of MST Target	Instrument used to quantify DNA or RNA in standards	Measured with a Nanodrop Lite (Thermo-Scientific, Wilmington, DE)
Quantification of MST Target	Evidence for absence of contaminating DNA	No template controls; extraction method blanks; field blanks
Quantification of MST Target	Definition of quantifiable replicate reactions	All replicates must be within 0.5 C1 of each other and will be averaged.
Quantification of MST Target	Evidence for absence of partial or complete inhibition	Internal amplification control spike, specifically, use of Sketa sample processing and inhibition control, in accordance with EPA Method B. Use of IAC in the HF183 PCR assay, in accordance with the SCCWRP - California Microbial Source Identification Manual.

Table S4. Laboratory Results for animal-host microbial source tracking marker concentrations and fecal indicator bacteria concentrations for the 11-hour study[a.]

Sampling Time	Location	Sample ID	Human (HF183 copies/100 mL)	Ruminant (Rum2Bac copies/100mL)	Dog (DogBact copies/100 mL)	Bird (LeeSeagull copies/100 mL)	*E. coli* (Colilert-18) MPN/100mL	Total Coliform (Colilert-18) MPN/100mL	Enterococci (Enterolert) MPN/100 mL
7:00	SITE 2A	W20-0987	ND	ND	ND	850	85	5,172	134
7:00	SITE 2B	W20-0988	ND	ND	BLOQ	ND	74	8,664	181
7:30	SITE 2A	W20-0989	ND	ND	ND	6,535	85	6,131	135
7:30	SITE 2B	W20-0990	ND	ND	ND	ND	120	7,701	158
8:00	SITE 2A R1	W20-0991	ND	ND	ND	579	75	9,208	171
8:00	SITE 2A R2	W20-0992	ND	ND	ND	734	86	6,867	173
8:00	SITE 2A R3	W20-0993	ND	ND	ND	1,229	75	3,609	122
8:00	SITE 2B R1	W20-0994	ND	ND	ND	ND	40	7,270	146
8:00	SITE 2B R2	W20-0995	ND	ND	BLOQ	ND	84	5,172	148
8:00	SITE 2B R3	W20-0996	ND	ND	ND	ND	171	6,488	148
8:15	SITE 1 R1	W20-0997	ND	ND	ND	BLOQ	31	9,208	20
8:15	SITE 1 R2	W20-0998	ND	ND	ND	BLOQ	52	6,488	<10
8:15	SITE 1 R3	W20-0999	ND	ND	ND	ND	52	8,164	51
8:15	SITE 1 R1	W20-1000	ND	ND	ND	BLOQ	20	8,164	<10
8:15	SITE 1 R2	W20-1001	ND	ND	ND	BLOQ	41	10,462	<10
8:15	SITE 1 R3	W20-1002	ND	ND	ND	BLOQ	20	10,462	41
8:30	SITE 2A	W20-1003	ND	ND	ND	5,340	86	5,794	187
8:30	SITE 2B	W20-1004	ND	ND	ND	ND	97	6,867	121
9:00	SITE 2A	W20-1005	ND	ND	BLOQ	2,293	148	7,701	187
9:00	SITE 2B	W20-1006	ND	ND	ND	ND	52	9,208	161
9:30	SITE 2A	W20-1007	ND	ND	BLOQ	10,827	132	6,867	315
9:30	SITE 2B	W20-1008	ND	ND	BLOQ	ND	84	5,794	187
10:30	SITE 2A	W20-1012	ND	ND	ND	408	110	5,172	109
10:30	SITE 2B	W20-1013	ND	ND	ND	ND	86	5,475	122
11:00	SITE 2A R1	W20-1014	ND	ND	ND	24,617	31	910	10
11:00	SITE 2A R2	W20-1015	ND	ND	ND	2,505	52	2,909	74
11:00	SITE 2A R3	W20-1016	ND	ND	ND	5,990	<10	3,654	52
11:00	SITE 2B R1	W20-1017	ND	ND	ND	ND	75	3,873	146
11:00	SITE 2B R2	W20-1018	ND	ND	ND	ND	84	3,255	160
11:00	SITE 2B R3	W20-1019	ND	ND	ND	ND	120	3,654	199
11:15	SITE 1 R1	W20-1020	ND	ND	ND	BLOQ	10	10	<10
11:15	SITE 1 R2	W20-1021	ND	ND	ND	ND	<10	20	<10
11:15	SITE 1 R3	W20-1022	ND	ND	ND	206	<10	10	<10
11:30	SITE 2A	W20-1026	ND	ND	ND	ND	41	1,467	86
11:30	SITE 2B	W20-1027	ND	ND	ND	ND	98	3,076	75
12:00	SITE 2A	W20-1028	ND	ND	ND	44,058	52	4,352	63
12:00	SITE 2B	W20-1029	ND	ND	ND	ND	109	4,352	75
12:30	SITE 2A	W20-1030	ND	ND	ND	ND	98	3,255	203
12:30	SITE 2B	W20-1031	ND	ND	ND	BLOQ	132	4,611	148
13:00	SITE 2A	W20-1032	ND	ND	ND	ND	<10	426	<10
13:00	SITE 2B	W20-1033	ND	ND	ND	ND	63	2,143	121
13:30	SITE 2A	W20-1034	ND	ND	ND	362	31	1,515	63
13:30	SITE 2B	W20-1035	ND	ND	ND	ND	52	2,247	62
14:00	SITE 2A	W20-1037	ND	ND	ND	2,695	41	4,884	52
14:00	SITE 2B	W20-1038	ND	ND	ND	541	41	5,172	75
14:30	SITE 2A	W20-1039	ND	ND	ND	1,078	31	6,131	85
14:30	SITE 2B	W20-1040	ND	ND	ND	209	72	6,131	86
15:00	SITE 2A R1	W20-1041	ND	ND	ND	383	86	3,654	52
15:00	SITE 2A R2	W20-1042	ND	ND	ND	1,168	52	6,867	63
15:00	SITE 2A R3	W20-1043	ND	ND	ND	718	63	5,794	98
15:00	SITE 2B R1	W20-1044	ND	ND	ND	BLOQ	41	6,488	41
15:00	SITE 2B R2	W20-1045	ND	ND	ND	BLOQ	62	6,867	74
15:00	SITE 2B R3	W20-1046	ND	ND	BLOQ	205	63	4,611	<10
15:15	SITE 1 R1	W20-1047	ND	ND	ND	2,798	<10	41	<10
15:15	SITE 1 R2	W20-1048	ND	ND	ND	2,033	<10	20	<10
15:15	SITE 1 R3	W20-1049	ND	ND	ND	1,330	<10	85	<10
15:30	SITE 2A	W20-1053	ND	ND	ND	13,835	884	9,208	119
15:30	SITE 2B	W20-1054	ND	ND	ND	BLOQ	122	6,131	63
16:00	SITE 2A	W20-1055	ND	ND	ND	8,355	97	4,884	52

Sampling Time	Location	Sample ID	Human (HF183 copies/100 mL)	Ruminant (Rum2Bac copies/100mL)	Dog (DogBact copies/100 mL)	Bird (LeeSeagull copies/100 mL)	*E. coli* (Colilert-18) MPN/100mL	Total Coliform (Colilert-18) MPN/100mL	Enterococci (Enterolert) MPN/100 mL
16:00	SITE 2B	W20-1056	ND	ND	ND	206	31	6,867	86
16:30	SITE 2A	W20-1058	ND	ND	ND	800	74	1,723	62
16:30	SITE 2B	W20-1059	ND	ND	ND	ND	73	2,481	31
17:00	SITE 2A	W20-1060	ND	ND	ND	BLOQ	63	2,909	109
17:00	SITE 2B	W20-1061	ND	ND	ND	BLOQ	41	2,187	86
17:30	SITE 2A	W20-1062	ND	ND	ND	4,272	131	3,448	110
17:30	SITE 2B	W20-1063	ND	ND	ND	ND	52	1,989	<10

[a]RED font indicates when the sample exceeded the single sample maximum limits. (ND = not detected; BLOQ = below limit of quantification; < = Lower Limit of Quantitation; R = replicate)

Table S5. Descriptive statistics of fecal indicator bacteria and the ROS (regression on order statistics) adjusted bird microbial source tracking marker in samples collected for the 11-Hr study at all sites combined.

	FIB (MPN/100ml)			MST (copies/100ml)
	E. coli	Total Coliform	Enterococci	Bird (ROS)
Number of Samples	66	66	66	66
Median	63	5,028	80	154
Mean	79	4,794	94	2,250
Standard Deviation	107	2,792	61	6,513
Coefficient of Variation	135%	58%	65%	290%
90 Percentile	125	8,857	183	6,154

Table S6. Summary of Spearman Rank correlations conducted between fecal indicator bacteria in samples collected at Clam Beach at Strawberry Creek and Clam Beach near Mad River.

		E. coli	Total Coliform	Enterococci
E. coli	Correlation Coefficient	1.000	**0.271***	**0.691****
	Sig. (2-tailed)	.	**0.028**	**0.000**
	N	66	**66**	**66**
Total Coliform	Correlation Coefficient	**0.271***	1.000	**0.314***
	Sig. (2-tailed)	**0.028**	.	**0.010**
	N	**66**	66	**66**
Enterococci	Correlation Coefficient	**0.691****	**0.314***	1.000
	Sig. (2-tailed)	**0.000**	**0.010**	.
	N	**66**	**66**	66

****. Correlation is significant at the 0.01 level (2-tailed). Considered strong association.**

***. Correlation is significant at the 0.05 level (2-tailed). Considered weak association.**

Table S7. Laboratory Results for animal-host microbial source tracking marker concentrations and fecal indicator bacteria concentrations [a].

Sampling Event	Location	Sample ID	Human (HF183 copies/100mL)	Ruminant (Rum2Bac copies/100mL)	Dog (DogBact copies/100mL)	Bird (LeeSeagull CAT copies/100mL)	*E. coli* (Colilert-18) MPN/100mL	Total Coliform (Colilert-18) MPN/100mL	Enterococci (Enterolert) MPN/100mL
1	SITE 1	W20-0461	ND	ND	ND	BLOQ	41	738	10
2	SITE 1	W20-0532	ND	ND	ND	ND	31	411	<10
3	SITE 1	W20-0566	ND	BLOQ	ND	BLOQ	31	801	20
4	SITE 1	W20-0637	ND	ND	ND	BLOQ	41	384	10
5	SITE 1	W20-0712	ND	ND	ND	ND	52	160	<10
6	SITE 1	W20-0761	ND	ND	ND	ND	20	700	20
7	SITE 1	W20-0787	ND	ND	ND	BLOQ	10	63	<10
8	SITE 1	W20-0876	ND	ND	ND	ND	52	172	<10
1	SITE 2A	W20-0462	ND	ND	BLOQ	ND	63	3,076	52
1	SITE 2A	W20-0484	ND	ND	ND	ND	63	3,448	10
1	SITE 2A	W20-0485	ND	ND	BLOQ	ND	41	3,448	41
2	SITE 2A	W20-0533	ND	BLOQ	ND	BLOQ	108	3,654	41
3	SITE 2A	W20-0567	ND	BLOQ	ND	ND	10	3,873	10
4	SITE 2A	W20-0638	ND	**769**	ND	ND	158	2,142	85
4	SITE 2A	W20-0639	ND	BLOQ	ND	ND	199	2,481	85
4	SITE 2A	W20-0640	ND	BLOQ	ND	ND	134	4,352	**110**
5	SITE 2A	W20-0713	ND	ND	ND	ND	<10	2,359	52

Sampling Event	Location	Sample ID	Human (HF183 copies/100mL)	Ruminant (Rum2Bac copies/100mL)	Dog (DogBact copies/100mL)	Bird (LeeSeagull CAT copies/100mL)	*E. coli* (Colilert-18) MPN/100mL	Total Coliform (Colilert-18) MPN/100mL	Enterococci (Enterolert) MPN/100mL
6	SITE 2A	W20-0762	ND	ND	ND	ND	20	2,613	63
7	SITE 2A	W20-0788	ND	BLOQ	ND	BLOQ	86	156	120
8	SITE 2A	W20-0877	ND	ND	ND	ND	<10	186	98
1	SITE 3	W20-0463	ND	ND	ND	ND	10	2,909	10
2	SITE 3	W20-0534	ND	BLOQ	ND	ND	63	5,172	63
3	SITE 3	W20-0568	ND	ND	ND	ND	20	3,873	31
4	SITE 3	W20-0641	ND	BLOQ	ND	ND	75	3,654	110
5	SITE 3	W20-0714	ND	ND	ND	ND	52	4,884	132
6	SITE 3	W20-0763	ND	ND	ND	ND	63	9,208	84
7	SITE 3	W20-0789	ND	BLOQ	ND	ND	63	209	52
8	SITE 3	W20-0878	ND	BLOQ	ND	ND	63	488	158
1	SITE 4	W20-0464	ND	ND	ND	ND	41	4,106	20
2	SITE 4	W20-0535	ND	BLOQ	ND	ND	31	2,851	20
2	SITE 4	W20-0536	ND	BLOQ	ND	ND	134	4,884	20
2	SITE 4	W20-0537	ND	**643**	ND	ND	146	3,873	30
3	SITE 4	W20-0569	ND	ND	ND	ND	20	3,441	52
4	SITE 4	W20-0642	ND	**588**	ND	ND	41	2,481	63
5	SITE 4	W20-0715	ND	ND	ND	ND	63	4,884	109
5	SITE 4	W20-0716	ND	ND	ND	ND	<10	2,046	63
5	SITE 4	W20-0717	ND	ND	ND	ND	31	2,143	146
6	SITE 4	W20-0764	ND	ND	ND	ND	41	5,475	75
7	SITE 4	W20-0790	ND	BLOQ	ND	ND	85	218	63
8	SITE 4	W20-0879	ND	BLOQ	ND	ND	30	290	98
1	SITE 5	W20-0465	ND	BLOQ	ND	ND	41	3,076	10
2	SITE 5	W20-0538	ND	ND	ND	ND	31	3,076	10
3	SITE 5	W20-0570	ND	ND	BLOQ	ND	52	5,172	31
3	SITE 5	W20-0571	ND	ND	BLOQ	ND	20	3,130	63
3	SITE 5	W20-0572	ND	ND	BLOQ	ND	96	3,076	10
4	SITE 5	W20-0643	ND	ND	ND	ND	63	4,352	63
5	SITE 5	W20-0718	ND	ND	**4,200**	ND	2,178	12,033	173
6	SITE 5	W20-0765	ND	BLOQ	ND	ND	181	1,100	203
6	SITE 5	W20-0766	ND	BLOQ	ND	ND	41	1,112	249
6	SITE 5	W20-0767	ND	ND	ND	ND	109	1,722	144
7	SITE 5	W20-0791	ND	ND	ND	ND	41	344	73
8	SITE 5	W20-0880	ND	ND	ND	ND	108	435	389
1	SITE 6	W20-0466	ND	ND	BLOQ	ND	20	1,266	31
2	SITE 6	W20-0539	ND	BLOQ	ND	ND	31	546	20
3	SITE 6	W20-0573	ND	ND	ND	ND	10	780	10
4	SITE 6	W20-0644	ND	**2,089**	ND	ND	97	839	41
4	SITE 6	W20-0645	ND	**1,581**	ND	ND	86	1,145	122
4	SITE 6	W20-0646	ND	BLOQ	ND	ND	122	1,081	41
5	SITE 6	W20-0719	ND	ND	ND	ND	41	480	122
6	SITE 6	W20-0768	ND	ND	ND	ND	20	4,352	52
7	SITE 6	W20-0792	ND	ND	ND	ND	160	712	132
7	SITE 6	W20-0793	ND	BLOQ	ND	ND	175	586	98
7	SITE 6	W20-0794	ND	ND	ND	ND	213	689	146
8	SITE 6	W20-0881	ND	BLOQ	ND	ND	52	785	173
1	SITE 7	W20-0467	ND	ND	ND	BLOQ	<10	2,282	41
2	SITE 7	W20-0540	ND	BLOQ	ND	ND	20	1,017	<10
3	SITE 7	W20-0574	ND	BLOQ	ND	ND	10	884	41
4	SITE 7	W20-0647	ND	**1,192**	ND	ND	121	1,850	62
5	SITE 7	W20-0720	ND	ND	ND	ND	41	1,076	134
5	SITE 7	W20-0721	ND	ND	ND	ND	52	959	31
5	SITE 7	W20-0722	ND	ND	ND	ND	41	1,050	110
6	SITE 7	W20-0769	ND	BLOQ	ND	ND	98	1,106	52
7	SITE 7	W20-0795	ND	ND	ND	ND	74	556	120
8	SITE 7	W20-0882	ND	**710**	ND	ND	75	780	171
8	SITE 7	W20-0883	ND	BLOQ	ND	ND	63	703	97
8	SITE 7	W20-0884	ND	**755**	ND	ND	52	789	201
1	SITE 8	W20-0468	ND	ND	ND	ND	30	2,909	52
1	SITE 8	W20-0486	ND	ND	ND	ND	63	2,046	52
1	SITE 8	W20-0487	ND	ND	ND	ND	20	2,143	135

Sampling Event	Location	Sample ID	Human (HF183 copies/100mL)	Ruminant (Rum2Bac copies/100mL)	Dog (DogBact copies/100mL)	Bird (LeeSeagull CAT copies/100mL)	*E. coli* (Colilert-18) MPN/100mL	Total Coliform (Colilert-18) MPN/100mL	Enterococci (Enterolert) MPN/100mL
2	SITE 8	W20-0541	**591**	ND	ND	ND	63	1,789	52
3	SITE 8	W20-0575	ND	ND	ND	ND	85	1,396	98
4	SITE 8	W20-0648	ND	ND	ND	ND	97	2,481	**336**
5	SITE 8	W20-0723	ND	ND	ND	ND	52	1,789	63
6	SITE 8	W20-0770	ND	ND	ND	ND	74	2,187	**161**
6	SITE 8	W20-0771	ND	ND	ND	ND	31	2,359	96
6	SITE 8	W20-0772	ND	ND	ND	ND	20	2,359	**121**
7	SITE 8	W20-0796	BLOQ	BLOQ	ND	ND	63	129	**146**
8	SITE 8	W20-0885	ND	ND	ND	ND	384	206	**246**
1	SITE 9	W20-0469	ND	ND	ND	ND	10	573	10
2	SITE 9	W20-0542	ND	ND	ND	ND	<10	583	31
2	SITE 9	W20-0543	ND	ND	ND	ND	20	602	10
2	SITE 9	W20-0544	ND	ND	ND	ND	20	581	51
3	SITE 9	W20-0576	ND	ND	ND	ND	31	809	**211**
4	SITE 9	W20-0649	ND	ND	ND	ND	20	538	63
5	SITE 9	W20-0724	ND	ND	ND	ND	<10	712	20
6	SITE 9	W20-0773	ND	ND	ND	ND	63	909	**146**
7	SITE 9	W20-0797	ND	ND	ND	ND	75	457	**181**
7	SITE 9	W20-0798	ND	ND	ND	ND	52	473	**148**
7	SITE 9	W20-0799	ND	ND	ND	ND	109	496	**146**
1	SITE 10	W20-0470	ND	BLOQ	ND	ND	20	1,250	31
2	SITE 10	W20-0545	ND	ND	ND	ND	31	754	10
3	SITE 10	W20-0577	ND	ND	ND	ND	10	538	<10
4	SITE 10	W20-0650	ND	BLOQ	ND	ND	109	743	75
5	SITE 10	W20-0725	ND	ND	ND	ND	31	754	52
6	SITE 10	W20-0774	ND	BLOQ	ND	ND	41	504	52
7	SITE 10	W20-0800	ND	ND	ND	ND	31	464	41
8	SITE 10	W20-0887	ND	ND	ND	ND	63	450	**132**
1	SITE 11	W20-0471	ND	ND	ND	ND	<10	573	<10
2	SITE 11	W20-0546	ND	ND	ND	ND	10	594	30
3	SITE 11	W20-0578	ND	ND	ND	ND	20	583	<10
3	SITE 11	W20-0579	ND	ND	ND	ND	<10	435	<10
3	SITE 11	W20-0580	ND	ND	ND	ND	10	743	10
4	SITE 11	W20-0651	**629**	ND	ND	ND	10	860	30
5	SITE 11	W20-0726	ND	ND	ND	ND	309	988	20
6	SITE 11	W20-0775	ND	ND	ND	ND	20	683	20
7	SITE 11	W20-0801	ND	ND	ND	ND	<10	169	20
8	SITE 11	W20-0888	ND	ND	ND	ND	10	313	31
8	SITE 11	W20-0889	BLOQ	ND	ND	ND	218	691	41
8	SITE 11	W20-0890	ND	ND	ND	ND	187	435	31
1	SITE 12	W20-0472	ND	BLOQ	ND	ND	31	1,250	<10
2	SITE 12	W20-0547	ND	ND	ND	ND	<10	364	20
3	SITE 12	W20-0581	ND	ND	ND	ND	<10	428	10
4	SITE 12	W20-0652	ND	**1,145**	ND	ND	144	886	63
5	SITE 12	W20-0727	ND	ND	ND	ND	10	855	**173**
6	SITE 12	W20-0776	ND	ND	ND	ND	20	573	**109**
7	SITE 12	W20-0802	ND	ND	ND	ND	73	457	10
8	SITE 12	W20-0891	ND	BLOQ	ND	ND	20	473	86
1	SITE 13	W20-0473	ND	ND	ND	ND	<10	1,430	<10
2	SITE 13	W20-0548	ND	ND	ND	ND	<10	384	<10
3	SITE 13	W20-0582	ND	ND	ND	ND	<10	364	<10
4	SITE 13	W20-0653	ND	BLOQ	ND	ND	10	328	52
5	SITE 13	W20-0728	ND	ND	ND	ND	86	**10,462**	**345**
6	SITE 13	W20-0777	ND	ND	ND	ND	20	408	20
7	SITE 13	W20-0803	ND	ND	ND	ND	122	420	41
8	SITE 13	W20-0892	ND	ND	ND	ND	31	443	**146**

[a]**RED** font indicates when the sample exceeded the single sample maximum limits. (ND = not detected; BLOQ = below limit of quantification; < = Lower Limit of Quantification)

Table S8. Descriptive statistics of fecal indicator bacteria in all samples collected for the 8-week study at all sites combined.

	FIB (MPN/100ml)		
	E. coli	**Total Coliform**	**Enterococci**
Number of Samples	135	135	135
Median	41	839	52
Mean	74	1,688	75
Standard Deviation	192	1,920	72
Coefficient of Variation	258%	114%	96%
90 Percentile	138	3,966	165

Table S9. A summary of each location, sample site characteristics and key findings of the 8-week study. Exceedance indicates when the sample exceeded the single sample maximum limits.

Site ID	Drainage Area & Land Use	Key Findings	Potential Source
Site 1	Ocean	• 4/8 Bird Detections (BLOQ)<br>• 1/8 Ruminant Detections (BLOQ)<br>• FIB - No Exceedances	Bird
Site 2a	-Drainage Area 7<br>-Ocean Site<br>-Public land use	• 5/12 Ruminant Detections (BLOQ)<br>• Ruminant Detection = 769 Copies/100 mL<br>• FIB – ENT Exceedance (2/12)	Ruminant
Site 3	-Drainage Area 6<br>-Strawberry Creek<br>-Public land use	• 4/8 Ruminant Detections (BLOQ)<br>• FIB - ENT Exceedance (3/8)	Ruminant
Site 4	-Drainage Area 6<br>-Strawberry Creek<br>-Public land use	• 4/12 Ruminant Detections (BLOQ)<br>• 2/12 Ruminant Detections = 588 & 643 Copies/100 mL<br>• FIB - ENT Exceedance (2/12)	Ruminant
Site 5	-Drainage Area 6<br>-Strawberry Creek & -Patrick Creek<br>-Public land use	• 3/12 Ruminant Detections (BLOQ)<br>• 3/12 Dog Detections (BLOQ)<br>• 1/12 Dog Detections = 4,200 Copies/100 mL<br>• FIB - ENT Exceedance (5/12)<br>• Collection Event 5 – All FIB Exceeded & 4,200 Copies/100 mL of Dog Detected	Dog
Site 6	-Drainage Area 6<br>-Strawberry Creek<br>-Public & medium density residential land use	• 4/12 Ruminant Detections (BLOQ)<br>• 2/12 Ruminant Detections = 1,588 & 2,089 Copies/100 mL<br>• 1/12 Dog Detections (BLOQ)<br>• FIB - ENT Exceedance (5/12)	Ruminant
Site 7	-Drainage Area 6<br>-Strawberry Creek<br>-Public & medium density residential land use	• 4/12 Ruminant Detections (BLOQ)<br>• 3/12 Ruminant Detections = 710, 750 & 1,192 Copies/100 mL<br>• 1/12 Bird Detections (BLOQ)<br>• FIB - ENT Exceedance (5/12)	Ruminant
Site 8	-Drainage Area 5<br>-Patrick Creek<br>-Public, agriculture, and low-density residential land use	• 1/12 Human Detections (BLOQ)<br>• 1/12 Human Detections = 591 Copies/100 mL<br>• 1/12 Ruminant Detections (BLOQ)<br>• FIB - ENT Exceedance (6/12)	Human
Site 9	-Drainage Area 4<br>-Patrick Creek<br>-Agriculture & medium density residential land use	• MST – No detections<br>• FIB - ENT Exceedance (5/12)	N/A
Site 10	-Drainage Area 3<br>-Strawberry Creek<br>-Medium density residential	• 3/8 Ruminant Detections (BLOQ)<br>• FIB - ENT Exceedance (1/8)	Ruminant
Site 11	-Drainage Area 3<br>-Strawberry Creek<br>-Medium density residential land use	• 1/12 Human Detections (BLOQ)<br>• 1/12 Human Detections = 629 Copies /100 mL<br>• FIB – No Exceedances	Human
Site 12	-Drainage Area 2<br>-Strawberry Creek<br>-Agriculture and timberland land use	• 2/8 Ruminant Detections (BLOQ)<br>• 1/12 Ruminant Detections = 1,145 Copies /100 mL<br>• FIB - ENT Exceedance (2/8)	Ruminant
Site 13	-Drainage Area 1<br>Rose Creek<br>Agriculture land use	• 1/8 Ruminant Detections (BLOQ)<br>• FIB - ENT Exceedance (2/8)<br>• FIB – TC Exceedance (1/8)	N/A

Table S10. Summary of master mix components and performance characteristics of each MST assay that includes Limit of Detection (LOD), Limit of Quantification (LOQ) and LOQ per 100 mLs of water filtered.

	**HF183**	**Rum2Bac**	**DogBact**	**Lee Seagull**
2x Master Mix	12.5 µl	12.5 µl	12.5 µl	10 µl
BSA	2.5 µl	2.5 µl	2.5 µl	2 µl
PCR Grade H_2O	3.5 µl	4.5 µl	7.4 µl	1.43 µl
Primer/Probe Mix	3.5 µl	3.5 µl	N/A	N/A
Forward/Reverse Primer	N/A	N/A	0.225 µl / 0.225 µl	0.63 µl / 0.63 µl
Probe	N/A	N/A	0.15 µl	0.31 µl
IAC Plasmid (500 copies)	1.0 µl	N/A	N/A	N/A
Total MM Volume	23 µl	23 µl	23 µl	15 µl
DNA Template	2 µl	2 µl	2 µl	5 µl
Final Volume	25 µl	25 µl	25 µl	20 µl
LOD	1 copy	1 copy	1 copy	1 copy
LOQ	10 copies / Reaction	10 copies / Reaction	10 copies / Reaction	10 copies / Reaction
LOQ/100 mls	500 copies	500 copies	500 copies	200 copies
